# Influence of flow regime on the decomposition of diluted methane in a nitrogen rotating gliding arc

**DOI:** 10.1038/s41598-022-14435-z

**Published:** 2022-07-09

**Authors:** Ananthanarasimhan J, Lakshminarayana Rao

**Affiliations:** Centre for Sustainable Technologies, Indian Institute of Science, Bengaluru, 560012 India

**Keywords:** Applied physics, Plasma physics, Engineering, Chemical engineering

## Abstract

This work reports the operation of rotating gliding arc (*RGA*) reactor at a high flow rate and the effect of flow regimes on its chemical performance, which is not explored much. When the flow regime was changed from transitional to turbulent flow ($5\rightarrow 50~\hbox {SLPM}$), operation mode transitioned from glow to spark type; the average electric field, gas temperature, and electron temperature raised ($106\rightarrow 156~\hbox {V}\cdot \hbox {mm}^{-1}$, $3681\rightarrow 3911~\hbox {K}$, and $1.62\rightarrow 2.12~\hbox {eV}$). The decomposition’s energy efficiency ($\eta _E$) increased by a factor of 3.9 ($16.1\rightarrow 61.9~\hbox {g}_{{\text{CH}}_{4}}\cdot \hbox {kWh}^{-1}$). The first three dominant methane consumption reactions (*MCR*) for both the flow regimes were induced by $\text {H}$, CH, and $\text {CH}_3$ (key-species), yet differed by their contribution values. The *MCR* rate increased by 80–148% [induced by *e* and singlet—$\text {N}_2$], and decreased by 34–93% [CH, $\text {CH}_3$, triplet—$\text {N}_2$], due to turbulence. The electron-impact processes generated atleast 50% more of key-species and metastables for every 100 eV of input energy, explaining the increased $\eta _E$ at turbulent flow. So, flow regime influences the plasma chemistry and characteristics through flow rate. The reported *RGA* reactor is promising to mitigate the fugitive hydrocarbon emissions energy efficiently at a large scale, requiring some optimization to improve conversion.

## Introduction

The emission of greenhouse gases cause climate change including global warming, an unavoidable problem due to dependence on fossil fuels^1^. Methane is the second largest contributor to global warming, contributed as much as $0.5\,^\circ$C since pre–industrial times^2^. Though $\text {CO}_2$ is the largest contributor, the global warming potential of $\text {CH}_4$ is 80 times that of $\text {CO}_2$ in the first 20 years after its release^2^; and its lifetime (decade) is shorter than $\text {CO}_2$ (century)^3^. For these reasons, mitigating $\text {CH}_4$ emissions at their sources is considered important which can lower temperatures quickly (due to 12 year response time) to prevent a temporary exceedance of the $2\,^\circ$C peak warming threshold (the goal of Paris agreement^3^). Globally, 40% of $\text {CH}_4$ emission comes from natural sources; the rest 60% comes from anthropogenic activities^4^—the sources easier for $\text {CH}_4$ mitigation^2^. Energy, industry, agriculture and waste sectors are the sources of anthropogenic methane emissions, with 50.63% and 20.61% of emissions are from agriculture and waste, respectively^4^. Particularly, in developing countries, activities such as open burning of biomass and agricultural residue (stubble burning), and landfills are one of the major contributors, also causing atmospheric pollution, affecting the human health and environment^4–7^—highlighting the problems and opportunities posed by $\text {CH}_4$.

Existing technologies for mitigation/decomposition/conversion of $\text {CH}_4$ include but not limited to thermo-, photo-, and biochemical conversion, with or without catalysts, as well as cascading of these technologies^8^. Recently, plasma technology which only require electricity as a source of energy, providing the possibility to use the intermittent excess renewable electricity^9^, is gaining interest for $\text {CH}_4$ conversion^10^, and fugitive $\text {CH}_4$ destruction/mitigation/decomposition^6^. Plasma is an ionized current-conducting gas consisting of ions, electrons, radicals, metastables, excited and neutral particles (together referred as *plasma species*), individually exhibiting multiple temperatures^11^. The plasma or plasma species are generated as follows: (1) an external electric field (*E*) is applied between the electrodes using a power source of desired specifications, and the space between the electrodes is filled with a gas to be treated; (2) the background free electrons present between the electrodes will accelerate due to the applied *E* and collide with the gaseous neutral particles; and (3) based on the energy exchanged during the collision, the atoms/molecules of the gas are either excited to higher energetic levels, or dissociated into neutral fragments/radicals, or ionized, forming a mixture of plasma species; (4) the continuous supply of energy input and sustained ionization results in the generation of avalanche of electrons that cause *breakdown*, striking an arc. Plasma can be used in the following two ways:

- as a source of heat (conventional thermal way) through temperatures of the order $10^3{-}10^4$ K provided by the *thermal plasmas*^12^; in thermal plasmas, temperatures of multiple components/species are in equilibrium (local thermodynamic equilibrium) raising the bulk gas to a very high temperature^11,13^.
- as a source of chemically active species even at ambient conditions without heating the bulk gas, provided by *non-thermal plasma (NTP)*^13^—preferred for chemical conversion applications (e.g. $\text {CH}_4$ conversion); in *NTP*, the average energy or temperature of the plasma species known to follow the order: electron temperature ($T_e$) > vibrational temperature ($T_V$) > rotational temperature ($T_r$) $\approx$ ion temperature ($T_i$) $\approx$ heavy neutral temperature ($T_o$) $\approx$ near room temperature ($T_{NR}$)^8,11,13^. The $T_o$ is the gas temperature ($T_{gas}$) of the plasma.

**Figure 1:**
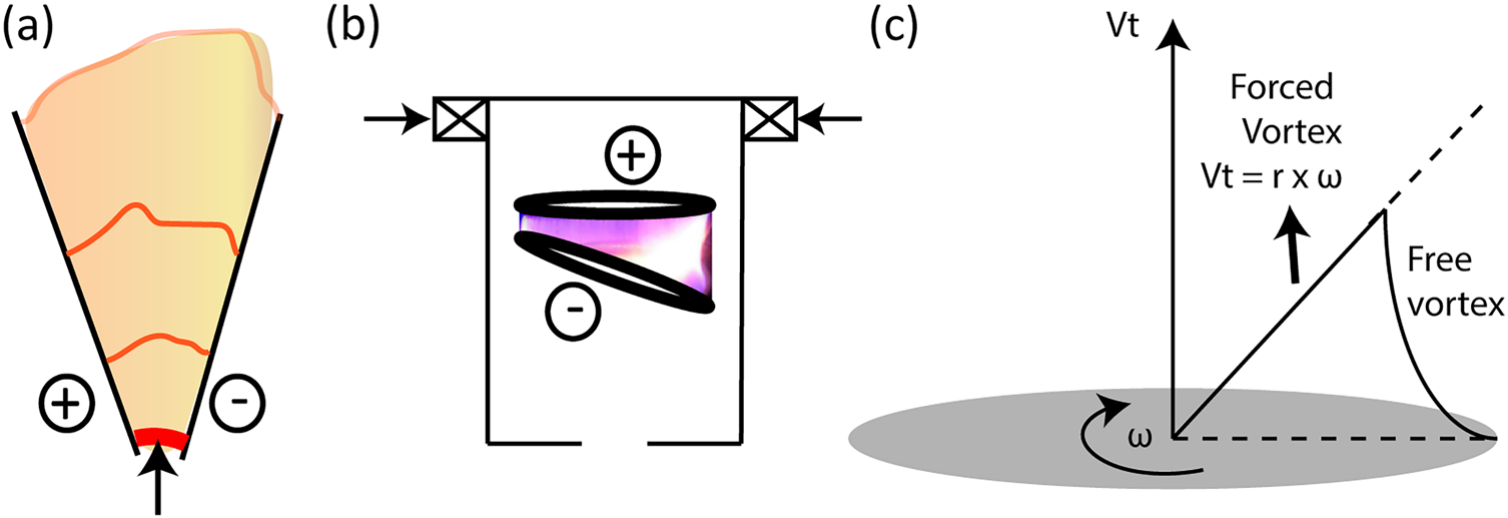
Schematic of (**a**) gliding arc discharge having planar diverging electrodes and (**b**) rotating gliding arc discharge of this work, showing the swept plasma volume; red thick streak near the shortest gap between the electrodes indicates initial breakdown discharge; arrows show the entry of gas to be treated by plasma. (**c**) sketch showing typical tangential velocity ($V_t$) profile in a swirl flow having forced vortex (linear profile) of constant angular velocity ($\omega$), and free vortex near the wall region.

*What is so special about NTPs?* Chemical reactions at a given temperature is limited due to uphill of $\Delta G$, and requires external energy as the input to overcome this uphill; for example, $\text {CH}_4$ would consume/require $75\,\hbox {kJ}\cdot \hbox {mol}^{-1}$ ($\approx 0.78\,\hbox {eV}\cdot \hbox {molecule}^{-1}$)^12,14^ to completely dissociate into C and $2\text {H}_2$. In thermo-chemical route, equilibrium temperature of $> 1500$ K is required for complete dissociation of $\text {CH}_4$, and atleast 600 K for indication of its dissociation. So, $\text {CH}_4$ dissociation/conversion is thermodynamically limited at ambient/room temperature. Whereas, in *NTP*, energetic plasma species can be generated even at ambient/room temperature, sufficient to overcome the uphill and dissociate $\text {CH}_4$—the speciality. Different types of *NTP* sources^15^ used for chemical applications include: glow/silent discharges, corona discharges, dielectric barrier discharge (*DBD*), microwave (*MW*) discharges, radio-frequency (*RF*) discharges, and gliding arc discharges (*GADs*). *GAD* is a blend of thermal and non-thermal plasmas, known as *warm plasmas*^16,17^ and can have high $T_e>1\,\hbox {eV}$, and high electron density ($n_e$) of $10^{13}$–$10^{15}\,\hbox {cm}^{-3}$, and $T_{gas}$ of the order of $10^3\,\hbox {K}$^1^. Traditionally, *GAD* has planar diverging electrodes providing 2D plasma volume [see Fig. 1a], limited by its poor arc–gas interaction, and narrow operating flow rates^13,18^. Various researchers including the authors addressed this by developing electrode configurations (retaining the diverging nature of the electrodes) that can provide 3D plasma volume^13^, known as *RGA*, like in this work [see Fig. 1b]. In *RGAs* tangential gas entry was used to create swirl flow which will force the struck arc to rotate and elongate simultaneously, achieving larger 3D plasma reaction volume compared to the traditional *GAD*. The arc rotation is also co-driven using external magnetic field^19^, referring the *RGA* as magnetically stabilized rotating gliding arc (*MRGA*)^13^.

Plasma source type is one of the major factors among many other that contributes to variability in the performance such as energy efficiency^10^. Warm plasmas are known to provide fast start and short transient time^20^; and provide 45% of the electrical energy to the endothermic reactions as in $\text {CH}_4$ conversion, and chemically efficient upto 40% (ratio of energy of reaction to the electricity consumed)^1^. For these reasons, *RGAs*/*MRGAs* are investigated for plasma-assisted chemical processes such as $\text {CH}_4$ conversion^21^. Typically, a gas mixture of $\text {CH}_4$ and $\text {CO}_2$ (as seen in biogas) is directly used as plasma forming gas, known as *dry reforming of methane (DRM)*^1,8^. Raja et al.^12^ used pure $\text {CH}_4$ as plasma forming gas, and investigated its conversion. In few other works, $\text {CH}_4$ was diluted in the inert gas such as argon^1,22^ and nitrogen^9^. Presence of $\text {N}_2$ helps to achieve stable plasma^9^, and the excited species and metastables of $\text {N}_2$ are reported to promote conversion of $\text {CH}_4$^23^. Further, most industrial emissions contain significant amounts of $\text {N}_2$ (mimicking the reality close enough) whose separation is costly^9^. Zhang et al.^16^ studied *MRGA*’s performance for $\text {CH}_4/\text {N}_2$ ratios in the range 0.05–1.6, and observed that the conversion of $\text {CH}_4$ decreased with the $\text {CH}_4/\text {N}_2$ ratio, attributing to the decreased $T_{gas}$.

Plasma reactors for the conversion of small molecules including $\text {N}_2$ and $\text {CH}_4$ is still at the laboratory scale^10^. The challenges of scaling up the plasma reactors to allow higher flow rates are still being explored and addressed^10^. Further, upscaling of plasma reactors are still considered largely empirical and difficult due to complexity of having reliable and scalable models of plasma reactors^10^. Nevertheless, to meet the growing scale of the demand for sustainable energy and chemistry, it is necessary to investigate the performance and behaviour at large flow rates ($>>1\,\hbox {LPM}$). Typically, low flow rates of the order mLPM are handled in *DBD*^24^ or corona or most of the *NTPs*; as higher gas flow rates are not favourable for energy efficient conversion^8^, probably due to plasma instabilities^20^. Among all the *NTP* sources including warm plasmas, *RGAs* are well adapted to scale-up for high flow rate applications^1,16^. Authors have previously designed a novel electrode configuration for *RGA*, having flexibility to scale up the plasma volume, without the necessity to increase the reactor size; and ever since, they have been exploring the gas–arc interaction effects on plasma behaviour^13,18,25^. Before presenting the objectives of this work, a related/relevant brief summary of contribution of authors in the past is presented in the next paragraph.

The gas and plasma dynamics are strongly coupled in *GAD*/*RGAs*^10,13,26^. The strong arc–gas coupling necessitates understanding the coupled effect on characteristics of the reactor; particularly, the effect of gas flow rate which is known to change the flow field [Reynolds number (*Re*)]^13,19^ and the physical parameters of the discharge^27^. However, the implications/effect of flow regimes or *Re* on characteristics or chemical performance is not fully explored or quantified in the existing works. A very few^19,28^, characterised *Re* based on the velocity of the gas calculated at the exit of the injector. The *Re* based on the injector exit velocity may not represent the gas–arc dynamics in the vicinity of the discharge. Indeed, the fluid velocity diminish axially downwards in a swirl flow^13^, and it is highly irregular in turbulent flow^29^. Authors, in their previous work, addressed this by defining the *Re* in the electrode region based on the average tangential velocity in the electrode region^13^. The basis/approach for defining the *Re* is given here for better clarity. In a given axial plane of the *RGA* reactor, the swirl flow is made of forced vortex and free vortex (near the wall), as shown in Fig. 1c. In such case, in the region of forced vortex, the typical tangential velocity increased linearly with the distance from axis of rotation^30^, having constant $\omega$ or gas rotational frequency ($f_{gas}$). In the free vortex flow near the wall region surrounding the forced vortex, the moment-of-momentum is conserved^30^. Since the free vortex is present only very near the wall, it can be ignored. So, the $\omega$ at a given plane is constant, from which average $V_t$ for a given plane can be calculated, based on which *Re* was defined. Like this, for applications involving rotating disk/pipe (rotational flows), Shevchuk et al. defined *Re* based on the angular velocity ($\omega$)^31,32^. Authors, in their very first work^13^, characterized the flow dynamics in the region between the electrodes for non-dimensional numbers such as (1) Reynolds number, and (2) Swirl number. They also quantified the angular velocity ($\omega$), $f_{gas}$, and arc rotational frequency ($f_{arc}$). These parameters were estimated for different flow rates (5, 25, and 50 SLPM), two different “number of tangential entry holes” (3 and 12), and at three axial planes between the electrodes. The flow regime (laminar, transitional, and turbulent) in the electrode region was obtained using the Reynolds number defined based on the tangential velocity ($V_t$) using the flow simulation. The defined Reynolds number was also found to have a linear relationship with the arc’s rotation; the arc rotation ($f_{arc}$) and gas rotation ($f_{gas}$) were comparable-validating the defined *Re*. Authors also reported the scaled velocity (ratio of velocity at the exit of tangential entries to the tangential velocity at the desired location/plane) for scaling purposes. Authors further demonstrated that the gas flow regime in the electrode region (based on the defined *Re*) influenced the electrical-, optical-, morphological-, and chemical characteristics of the $\text {N}_2$–*RGA*^26^; particularly, at highly turbulent flows ($\hbox {Re}\ge 10^{4}$), the eddies of Kolmogorov length were found to be smaller than the arc diameter, and hence could penetrate and distort/shear the discharge (morphological), increasing the plasma’s heat and mass transport rate to the surroundings by turbulent convective mixing (Péclet number $>1$)—indicating a strong coupling of gas and arc dynamics^26^; the eddies also caused reignition events, and spatial inhomogeneity of charges, affecting the *E*^26^; the affected *E* (electrical) influenced the collisional processes (optical, and chemical), which eventually changed the plasma properties^26^.

This work further reports the effect of transitional (5 SLPM) and highly turbulent flow regime (50 SLPM) on the decomposition of diluted $\text {CH}_4$ in $\text {N}_2$–*RGA* (mimicking fugitive emissions), having $CH_4/\text {N}_2$ ratio of $\approx 0.01$ (1% of $CH_4$ by volume). Existing works in *RGAs* for $\text {CH}_4$ conversion explored the flow rates of 1–10 LPM^1,9,12^; the maximum of 24 LPM was studied by Zhang et al.^16^, as mentioned earlier. So, a flow rate of 50 SLPM for turbulent flow was chosen having a *Re* of the order $10^4$, which is not yet explored in the literature. The $\text {CH}_4/\text {N}_2$ ratio of 0.01 was chosen from the fundamental research point of view to understand the effect of highly turbulent flow. The effect of change in the flow regime between transitional and turbulent flow was observed on the average reduced electric field $\left( \frac{E}{N}\right)$ and $T_{gas}$, and the $\eta _E$ in $\text {CH}_4$ conversion. Further, the chemical kinetics simulation was performed for the same experimental conditions to understand the dominant reactions involved in consumption of $\text {CH}_4$ at transitional and turbulent flow regimes.

## Materials and methods

### Details of the electrode configuration, RGA reactor, and its operation

**Figure 2:**
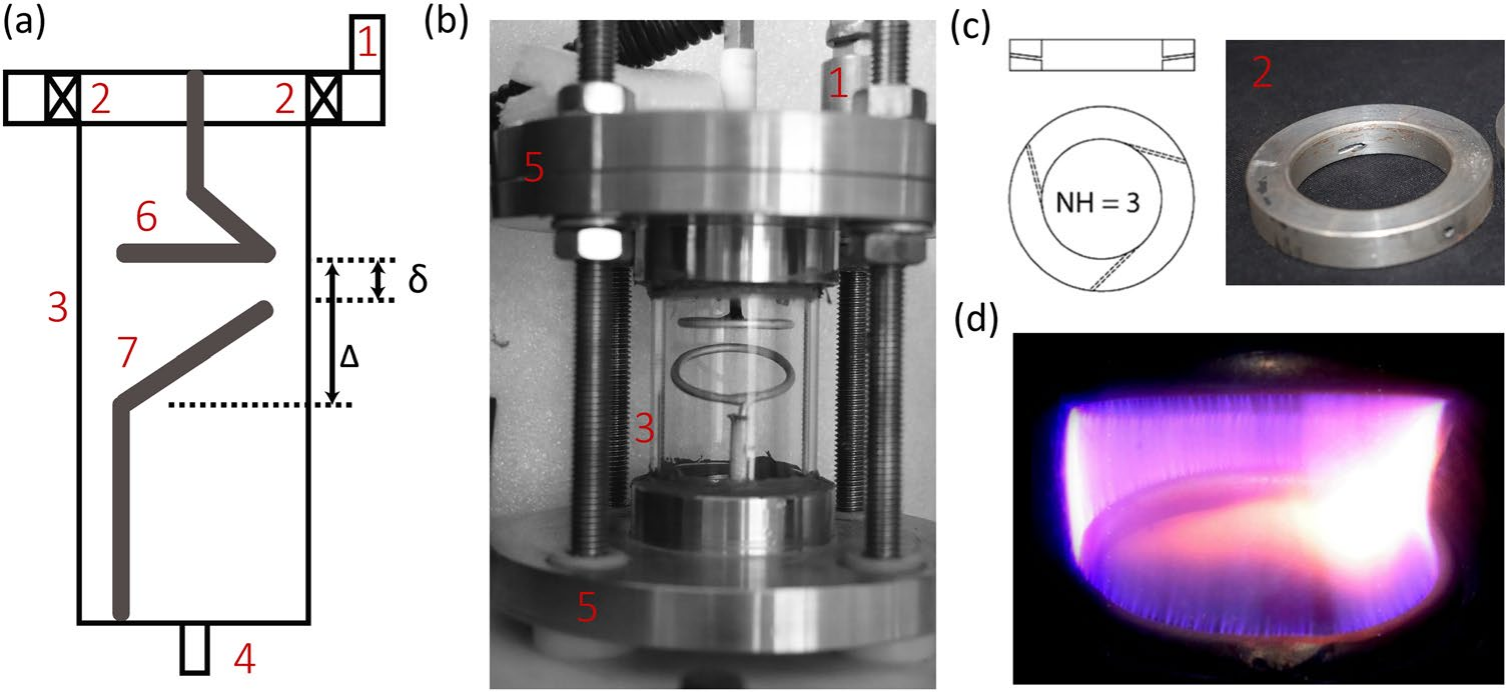
Schematic (**a**) and assembled setup (**b**) of the *RGA* reactor with the components numbered: 1—gas inlet, 2—swirl disc, 3—Quartz reactor, 4—gas outlet, 5—steel flanges, 6—high voltage electrode and 7—ground electrode; (**c**) swirl disc with three tangential gas entry holes (Number of Holes, $\hbox {NH} = 3$); (**d**) snapshot of a rotating arc forming a swept plasma volume (1% $\text {CH}_4$ in $\text {N}_2$).

Figure 2a,b shows the schematic and assembled setup of the *RGA* reactor, respectively, indicating the functional parts numbered 1 through 7. The reactor wall was made of Quartz cylinder of inner diameter (*D*) of 40 mm, and height 80 mm height. Quartz reactor was closed at both the ends by steel flanges, and a 5 mm gas inlet and outlet were provided in the top and bottom steel flanges, respectively. The electrode configuration consisted of flat and inclined aluminium rings housed inside the quartz cylinder, creating a minimum and a maximum gap between the electrodes, referred as $\delta$ (3 mm) and $\Delta$ (14 mm), respectively. The diameter of flat ring, and the projected horizontal length of inclined ring, both was 30 mm. This arrangement facilitates easy selection of (1) axial position of the electrodes, and (2) inter electrode gap between the electrodes by varying the axial position of either of the electrodes. The gas fed to the main inlet entered the quartz reactor through three tangential entry holes of 1.6 mm diameter, provided in the swirl disc [see Fig. 2c], creating a gas vortex or swirl flow. When breakdown *E* was achieved between flat/high-voltage and inclined/ground electrodes, a discharge struck near $\delta$. Influenced by the gas–vortex, the struck arc started to rotate. The rotating arc elongated during the first half of its rotation and then contracted during the second half, forming a 3D plasma volume [see Fig. 2d].

### Experiments

#### Flow rates studied

The objective of this work is to study the effect of flow regime on the characteristics and performance of the conversion of diluted methane in nitrogen–*RGA*. The *Re* that indicates the flow regime was defined by the authors in their earlier work for the region between the electrodes as^13^,

1 $$\begin{aligned} Re = \frac{V_tD}{\nu }, \end{aligned}$$

where $V_t$ is the average area-weighted tangential velocity in $\hbox {m}\cdot \hbox {s}^{-1}$ in the region between the electrodes, $\nu$ is the kinematic viscosity in $\hbox {m}^{2}\cdot \hbox {s}^{-1}$ at ambient conditions of the feed gas. Based on this definition, authors have calibrated and established the flow regimes of the flow rates for argon, nitrogen and oxygen in their previous works^13,18,25,26^. Calibration of *Re* for a flow rate of any gas can be obtained using the procedure established by the authors^13^ as follows: (1) obtain $f_{arc}$ by visual observation using high-speed camera (*HSC*) or by applying FFT on discharge voltage (*V*); (2) obtain $V_t$ using the linear relation between $f_{arc}$ and $f_{gas}$ observed for this *RGA* and reported by authors in their earlier work^13^, which is,

2 $$\begin{aligned} f_{gas} = f_{arc} = \frac{V_t}{\pi D}; \end{aligned}$$

(3) feed $V_t$ in Eq. (1) and calculate *Re*.

Authors verified that Eq. (2) is applicable to obtain *Re*, using multi-methods approach including cold flow simulation, and reported in their previous work^13^. Based on these steps, the transitional and turbulent flow regimes were observed for 5 and 50 SLPM, respectively, chosen as the flow rates of this study. The flow rates corresponding to laminar flow ($< 5\,\hbox {SLPM}$) caused poor rotation, and hence ignored, as seen and reported by the authors in their previous works^13,25^. The calculated *Re* will be discussed in “Influence of flow regimes on plasma characteristics (average estimates)” section.

#### Gas mixture chosen

A gas mixture of $\text {CH}_4$ (1%) and $\text {N}_2$ was supplied in a cylinder by M/s Chemix Specialty Gases and Equipment, and the concentration of methane was tested and certified with an accuracy of $\pm 1\%$. A $\text {CH}_4$ concentration of 1% ($\text {CH}_4/\text {N}_2$ ratio of 0.01) by volume was chosen for the following reasons:

- Landfill gas at the “after care” stage is reported to have $\text {CH}_4$ concentration $< 3\,\%$, having major components as $\text {CH}_4$ and $N_2$ (if directly pumped)^6^.
- It is acceptable to use high dilution for the purpose of scientific work like this work which focused on investigating the effect of flow regime on $\text {CH}_4$ conversion; Kong et al.^33^ investigated the effect of high pressure on the layered structure surrounding the discharge alone, in a $\text {CH}_4{-}\text {N}_2$–*GAD*, at a very high dilution of 0.1% by volume.
- The lowest $\text {CH}_4/\text {N}_2$ ratio studied in the literature is 0.05 by Zhang et al.^16^; Zhang reported that the methane conversion decreased with the $\text {CH}_4/\text {N}_2$ ratio in the range 0.05–0.6; and slight improvement was observed only $>0.6$, which is much higher for the objective of this work; so the authors wanted to explore a ratio below 0.05.

### Experimental run

**Figure 3:**
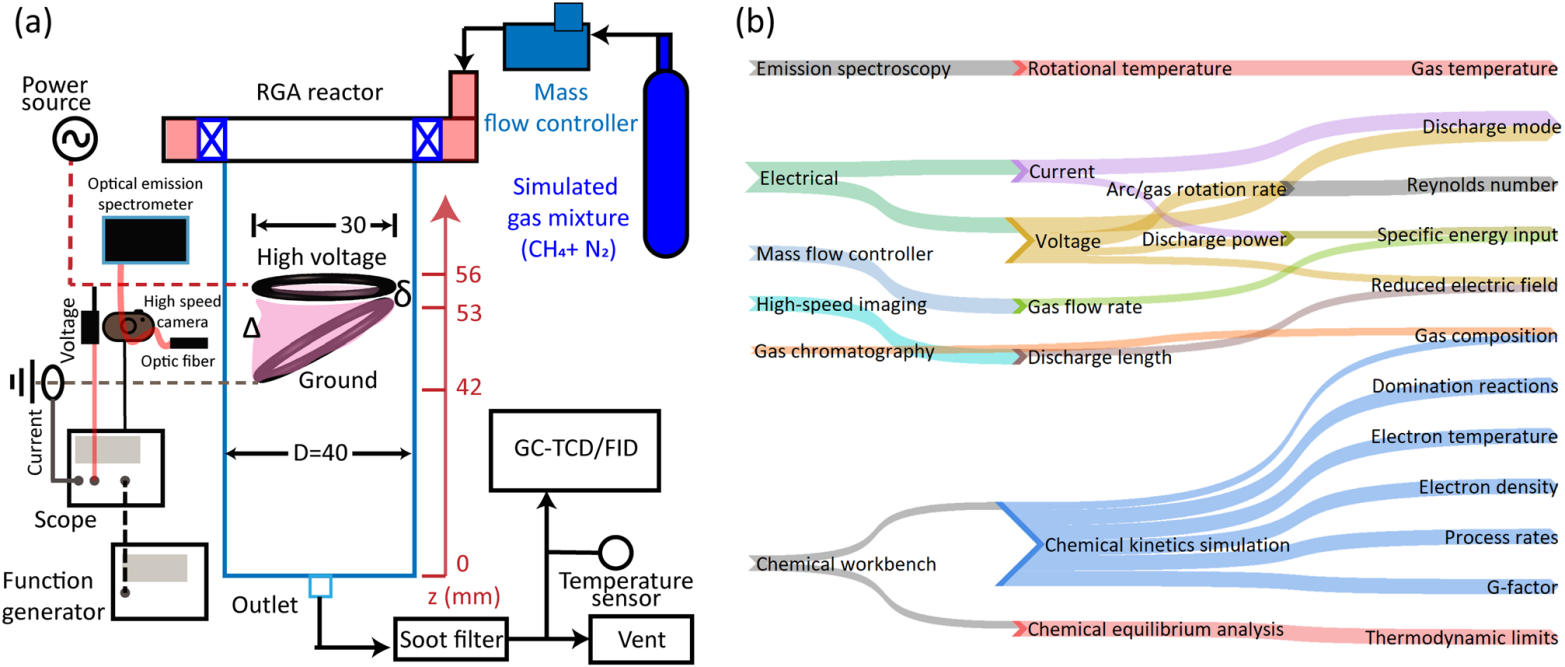
(**a**) Schematic of experimental/diagnostic setup; all dimensions are in mm. (**b**) Schematic flow from diagnostic/simulation tools to the analysis parameters.

Figure 3a shows a schematic of the experimental/diagnostic setup. The gas mixture was fed to the *RGA* at the desired operating flow rates controlled by a mass flow controller. Once the gas was fed to the *RGA*, plasma was switched ON. The voltage level in the power supply was set constant for both the flow rates. Typically, after 5–6 min of operation, the data collection was performed including the online gas sampling for product gas composition analysis, which will be detailed in “Methodology-experiments” section. The run time was decided after verifying initially that the composition of the products were stabilized before taking the data collection. Before each experiment run, the reactor and the pipe lines were flushed using argon and ensured that all the traces of previous experiment were removed, which was ensured by no gas peaks in the gas chromatography (*GC*). The *RGA* was also once operated for more than 30 min at 5 LPM; the smooth rotation and nearly constant power input to the discharge (*P*) indicated stable operation of the *RGA*; a very trace soot deposition was observed as the dilution is very high, and a major fraction of the soot would be flushed out due to the reactor design and electrode configuration, specifically at 50 SLPM. Each experimental condition was repeated thrice at minimum, and the observations were verified for reproducibility.

### Methodology-experiments

Figure 3b shows the schematic describing the diagnostic/simulation tools used to determine parameters/observations. The effect of flow regime was captured by determining the change in the average value of the representative parameters of the plasma, namely $T_{gas}$, $T_e$, and $\frac{E}{N}$; and the performance parameters such as $\text {CH}_4$ conversion, and $\eta _E$ by determining the product gas composition. These parameters were determined using diagnostic tools such as *HSC*, optical emission spectroscopy (*OES*), voltage–current ($V{-}I$) probes, and *GC* as shown in Fig. 3a. The equipment specifications used is given in Table 1.

**Table 1:** Details of the equipment used in the diagnostic setup.

Equipment	Make/model, specification
Mass flow controller	Alicat Scientific
Power source	M/s Information Unlimited
	PVM500, 20 kV peak, $\approx 19\,\hbox {kHz}$
Voltage, current probe	Tektronix P6015A, TCP312A
Oscilloscope	Tektronix TBS2074
	–Bandwidth:70 MHz
	–Record length: 2M points
High-speed camera	Kron technologies Chronos1.4
Lens	Computar 12 mm, f/1.4
Optical emission spectrometer	Ocean Insight HDX
	–Optical resolution: 0.6–0.72 nm
	–Slit width: $10\,\upmu \hbox {m}$
	–Fiber diameter: $600\,\upmu \hbox {m}$
	–Acceptance angle: $25.4^\circ$
Gas chromatography	M/s Mayura Analytical

#### Electrical measurements

Electrical probes and oscilloscope were used to capture $V{-}I$ waveforms measured for the duration *T*. The *P* expressed in W for an operating condition was calculated using the expression^18^

3 $$\begin{aligned} P = \frac{1}{T}\int _{0}^{T}{VI dt}. \end{aligned}$$

The *V* waveform was also used to calculate $f_{arc}$ in Hz by applying FFT on *V* as detailed by authors in their previous work^13^. Further, using $f_{arc}$, *Re* and $f_{gas}$ were obtained due to their linear relation shown by the authors in their previous work^13^ as shown in Eq. (2).

#### Optical emission spectroscopy

The visible emission spectrum from the *RGA* was captured using an absolutely calibrated *OES* and optic fiber detailed in Table 1. The optic fiber supported by collimator was positioned to capture the light anywhere from the plasma region whose position kept changing due to its rotation. The acquisition time was set to 0.5 s to ensure that the captured spectrum represented *RGA*’s average emission characteristics. The $C_2$ Swan band between 480 and 520 nm, was fitted using SPECAIR tool^34,35^, to obtain $T_r$ as per the most commonly practiced technique^16,36,37^. The obtained $T_r$ was considered $T_{gas}$ due to their equilibration expected in atmospheric pressure plasmas like *RGAs*^26,36^.

#### High-speed imaging

Arc images captured using the *HSC* was used to obtain the discharge length ($l_d$) of the *RGA*. The working distance between the camera and the object was set to 150 mm, achieving a resolution of $0.09\,\hbox {mm}\cdot \hbox {pixel}^{-1}$. The frame capture rate was set to 16–19 kHz, optimized to cover the entire discharge zone for the working distance of 150 mm, the minimum provided by the used prime lens. Images for $l_d$ measurement were captured at an exposure of $10\,\upmu \hbox {s}$. The $l_d$ is the projected arc length measured using *regionprops* technique, reported by authors in their previous work^38^ including the capture settings. Given the complexity involved in measuring the 3D length, using the projected length for analysis and estimation of derived parameters is considered an accepted practice^19,27,38,39^.

#### Reduced electric field (E/N) estimation

The average $\frac{E}{N}$ was estimated using the following steps, the methodology used by the authors earlier^18^ and by Kong et al.^27^: (1) capture synchronized the electrical signals and high-speed images; (2) calculate the “$l_d$” of the discharge in every image-frames captured using the *HSC*; (3) estimate the $V_{RMS}$ corresponding to every image-frames; the *V* data corresponding to the time duration between the start and stop of the *HSC*’s exposure in every frame was used to calculate $V_{RMS}$; (4) fit a linear function on the $l_d$ vs. $V_{RMS}$ to obtain the average *E* which is the slope of the linear fit; (5) obtain $T_{gas}$ using the *OES*, and calculate the number density of neutral particle in its ground state (*N*); (6) take the ratio of the average *E* and *N*.

#### Gas chromatography

The gaseous products were analyzed using the *GC* (M/s Mayura Analytical). *Sampling procedure* The product at the outlet of the *RGA* in the gaseous form was split into two; one was fed through the soot/particle collector to the 1 ml sampling loop of an online *GC* for quantification; the other was vented out. The gas in the sampling loop at the end of operation time (5–6 min) was analysed by the *GC*. *Quantification procedure* The thermal conductivity detector (*TCD*) was used to quantify $\text {H}_2$ and $\text {N}_2$, and flame ionization detector (*FID*) was used to quantify hydrocarbons such as $\text {CH}_4$, $\text {C}_2\text {H}_2$, $\text {C}_2\text {H}_4$, and $\text {C}_2\text {H}_6$. The temperature of the outlet gas entering the sampling loop of the *GC* was $25 \pm 2\,^\circ \hbox {C}$, measured using a temperature sensor. Due to the fact that both the inlet and outlet gas were at near ambient condition, the gas composition was estimated without gas-expansion correction, like Zhang et al.^16^. A Hayesep-A column ($2\,\hbox {m} \times 3\,\hbox {mm}$), and a Zeolite molecular sieve column ($2\,\hbox {m} \times 3\,\hbox {mm}$) was used in series to achieve efficient separation of the species, setting the column/oven temperature at $60\,^\circ \hbox {C}$, choosing argon as the carrier gas. With the same settings, the *GC* was calibrated for wide range of concentrations of the species relevant to this work using reference calibrations gas mixtures (M/s Chemix Specialty Gases and Equipment). A random calibration was also performed right before the experimental run, to verify the consistency, and any slight changes occurred were accounted and updated to ensure accurate quantification.

The outlet gas flow rate was calculated using the inlet molar flow rate of $\text {N}_2$, by considering $\text {N}_2$ as non-reacted in the process^40^. The *P* in *W* was calculated using Eq. (3). The $\eta _E$ in $\hbox {g}\cdot \hbox {kWh}^{-1}$, the carbon balance ($\text {C}_{balance}$) and $\text {CH}_4$ conversion (${\text {C}}_{\text {CH}_4}$) in % were calculated using the expressions as follows:

4 $$\begin{aligned} \eta _E= & {} \frac{36\times 10^{5}\times ({\text {CH}}_4,_{in}{-}{\text {CH}}_4,_{out})}{P}, \end{aligned}$$

5 $$\begin{aligned} {\text {C}}_{balance}= & {} \frac{{\dot{C}}_{out}}{{\dot{C}}_{in}} \times 100, \end{aligned}$$

6 $$\begin{aligned} {\text {C}}_{{\text {CH}}_4}= & {} \frac{[{\text {CH}}_4]_{in}-[{\text {CH}}_4]_{out}}{[{\text {CH}}_4]_{in}} \times 100. \end{aligned}$$

The $[CH_4]_{in~or~out}$ is the molar flow rate of methane in mol$\cdot$s^−1^, ${\dot{C}}_{in~or~out}$ is the mass flow rate of the Carbon atom in g$\cdot$s^−1^, and $\text {CH}_4,_{in~or~out}$ is the mass flow rate of methane in $\hbox {g}\cdot \hbox {s}^{-1}$, at the inlet and outlet conditions.

### Methodology—chemical kinetics simulation

**Figure 4:**
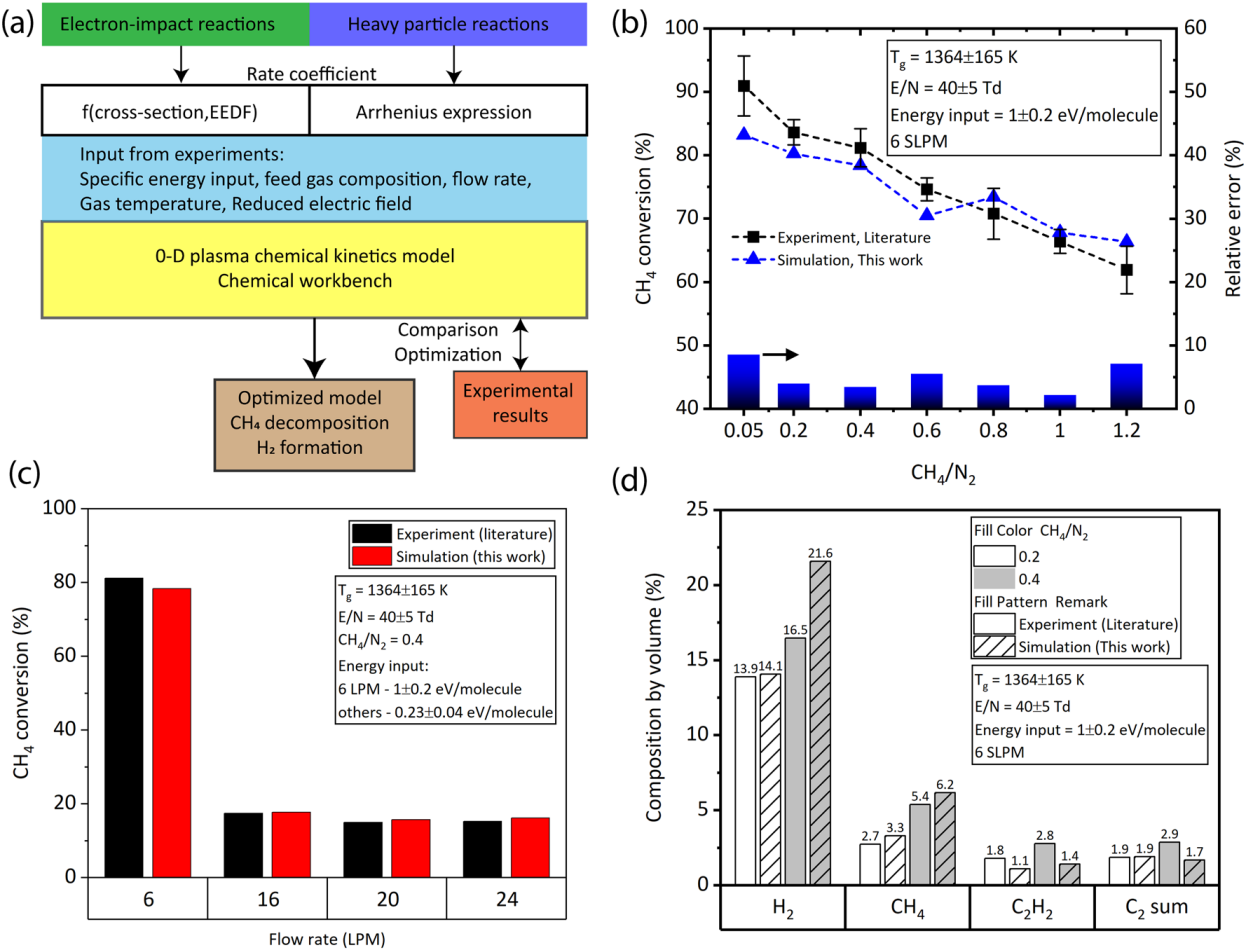
(**a**) Schematic of the procedure for the chemical kinetics simulation. Validation of the mechanism simulated using the model/approach in this work with the experimental results from literature: $\text {CH}_4$ conversion as a function of (**b**) $\text {CH}_4/\text {N}_2$ ratio, and (**c**) flow rate; (**d**) composition of selected species in the product at $\text {CH}_4/\text {N}_2$ ratio of 0.2 and 0.4, at 6 SLPM.

To elucidate the chemistry of $\text {CH}_4$ conversion, chemical kinetic simulation and thermodynamic equilibrium analysis was performed using the tool Chemical Workbench. Figure 4a shows the schematic of the procedure adapted for the 0-D chemical kinetics simulation. The VIBRKIN reactor module of Chemical Workbench software, a well-developed and proprietary software of M/s Kintech Laboratory^41^ was used to solve the non-equilibrium plasma reactions together with the heavy particle reactions^26,42–45^. A total of 81 species were included in the model, which react to each other through 274 reactions, detailed in Table SI of the Supplementary Material. The dominant electron-impact reactions such as momentum transfer, electronic-, vibrational-, rotational-excitation, dissociation and ionization processes of the main species i.e., $\text {N}_2$, $\text {H}_2$, $\text {CH}_4$, CH, $\text {CH}_2$, $\text {CH}_3$, $\text {C}_2\text {H}$, $\text {C}_2\text {H}_2$, $\text {C}_2\text {H}_3$, $\text {C}_2\text {H}_4$, $\text {C}_2\text {H}_5$, and $\text {C}_2\text {H}_6$ were considered. The rate coefficients of electron induced chemical reactions was calculated based on numerical solution of Boltzmann kinetic equation for electron energy distribution function (*EEDF*). The cross-sections of corresponding plasma reactions were taken from the published databases (Table SI in Supplementary Material). Heavy particle species including excited species of $\text {N}_2$, H, $\text {H}_2$, $\text {C}_mH_n$ ($1\le \hbox {m}\le 3$, $0\le \hbox {n}\le 2\hbox {m} + 2$), and neutral nitrogen species such as N, HCN, CN, NH, $\text {NH}_2$, $\text {NH}_3$ were considered in the model. Literature works^23,46^ experimentally detected intermediate product *CN*, and gas products HCN and $\text {NH}_3$. In this work, the typical emission spectrum of $\text {CH}_4+N_2$ plasma at operating conditions was dominated by the CN violet system ($B^2\Sigma \rightarrow X^2\Sigma$) band was observed, with the maximum intensity at $\approx 388\,\hbox {nm}$ (0,0)—agreeing with the observations of Zhang et al.^16^. Therefore the reactions involving neutral $\text {N}_2$—containing species been included in the mechanism. For heavy particle reactions the rate coefficients were given in terms of coefficients of Arrhenius expression, adopted from NIST database and other literature work^46^. The ion involved heavy particle reactions were not considered, as the *RGAs*’ average $T_e$ is typically low, in the range of 1–2 eV^26,46^, also the $T_e$ value seen in this work, discussed in “Influence of flow regimes on plasma characteristics (average estimates)” section. The *RGA* reactor was represented in the Chemical Workbench using the $\frac{E}{N}$, $T_{gas}$ and the specific energy input (*SEI*) obtained from the experiments as input parameters, like in the previous work of the authors^26^. Instead of gas residence time, the *SEI* was considered as the reaction time limiting factor; because, (1) the calculation of the discharge volume to estimate the gas residence time for highly complex *RGA* is challenging, (2) the gas residence time experienced by the particles flowing between the electrodes is reported to have wide distribution^47^, and (3) the discharge is instantaneously located at a given position, and changes its volume during its rotation. The *SEI* was calculated assuming that the entire molecules at a given flow rate was assumed to experience the applied *E*, expressed in the unit of $\hbox {eV}\cdot \hbox {molecule}^{-1}$. The chemical mechanism was built based on the mechanism available in the literature for $\text {CH}_4{-}\text {N}_2$–*RGA*^46^, and further optimized after comparing the simulation results with the experiments, by addition of reactions or by using different choice of cross-section and rate coefficients. The rate coefficients together with the corresponding references are provided in the Supplementary Material. The following parameters were obtained from Chemical Workbench:

1. G-factor of the species; G-factor is the number of molecules of a species generated per 100 eV of energy consumed^48,49^;
2. process rates in ($\hbox {cm}^{3}\cdot \hbox {s}^{-1})^{-1}$;
3. composition of the species at the outlet in ppmV;
4. $$n_e$$ in $$\hbox {cm}^{-3}$$; and
5. $$T_e$$ in eV.

Before using this model for comparison with the experiments of this work, the mechanism/model was validated by performing simulation for the experiments performed by Zhang et al.^16^, by giving the input conditions of their work. Specifically, the trend in predicting the $\text {CH}_4$ conversion at different flow rates, and $\text {CH}_4/\text {N}_2$ ratios were validated, along with checking for fair agreement in predicting the products’ composition such as ${\text {H}}_2$ and $\text {C}_2$ hydrocarbons. For the sake of readability, the results of the validation are discussed here in methodology, in “Validation of the model and mechanism” section.

#### Validation of the model and mechanism

Figure 4b,c show the comparison of simulated (this work) and experimental (literature^16^) $\text {CH}_4$ conversion as a function of $\text {CH}_4/\text {N}_2$ ratio, and flow rate, respectively. A good agreement was seen for $\text {CH}_4$ conversion Vs. $\text {CH}_4/\text {N}_2$ ratio (relative error $< 8.6\%$), and for $\text {CH}_4$ conversion Vs. flow rates (relative error $< 6\%$). Additionally, the outlet gas compositions of the simulated and the experimental was compared, shown in Fig. 4d, indicated fair agreement. The results clearly indicated that the model approach and the mechanism adapted in this work is applicable for a wide range of operating conditions.

In view of plasma chemistry, good agreement is acceptable^46^, and therefore can be used further to understand the underlying reaction mechanisms and pathways, in the conversion of diluted $\text {CH}_4$ in $\text {N}_2$–*RGA*.

#### Thermodynamic analysis

The thermodynamic equilibrium reactor module available in the chemical workbench software was used to estimate the $\eta _E$ achieved by thermodynamic equilibrium, the thermodynamic limit for this work’s conditions i.e., transitional and turbulent flow. The $T_{gas}$ corresponding to the flow regimes was given as the input, along with the feed mixture condition (1% $\text {CH}_4$, rest $\text {N}_2$) as input to the thermodynamic equilibrium reactor.

## Results and discussion

### Influence of flow regimes on plasma characteristics (average estimates)

Table 2 shows the parameters obtained for the transitional (5 SLPM) and turbulent (50 SLPM) flow regimes. As it was observed in the previous works^25,26^, based on *VI* waveforms, the discharge mode was glow-type at transitional, and spark-type at turbulent. When changed from transitional to turbulent flow, the average *E*, $\frac{E}{N}$, $T_{gas}$, $T_e$ increased by 46%, 24%, 6%, and 31%, respectively. The *SEI* dropped by 64%, because the *P* increased only by 91% ($\approx 24\,\hbox {W}\rightarrow \approx 45\,\hbox {W}$), whereas the flow rate increased by 900%. The $n_e$ between the flow regimes were comparable of same order, with small difference ($\approx 7.6\%$).

**Table 2:** Plasma characteristics at the operating conditions.

Flow rate (SLPM)	Flow regime	*Re* ($\times 10^4$)	*SEI* ($\hbox {eV}\cdot \hbox {molecule}^{-1}$)	Discharge mode	*E* ($\hbox {V}\cdot \hbox {mm}^{-1}$)	$\frac{E}{N}$ (Td)	$T_{gas}$ (K)	$T_e$ (eV)	$n_e$ ($10^{18}\,\hbox {m}^{-3}$)
5	Transitional	0.36	0.07 ± 0.01	Glow	$\approx 106$	$\approx 82$	$3681 \pm 261$	$\approx 1.62$	3
50	Turbulent	$5.4 \pm 0.2$	0.025 ± 0.01	Spark	$\approx 156$	$102 \pm 3$	$3911\pm 290$	$\approx 2.12$	2.77

**Figure 5:**
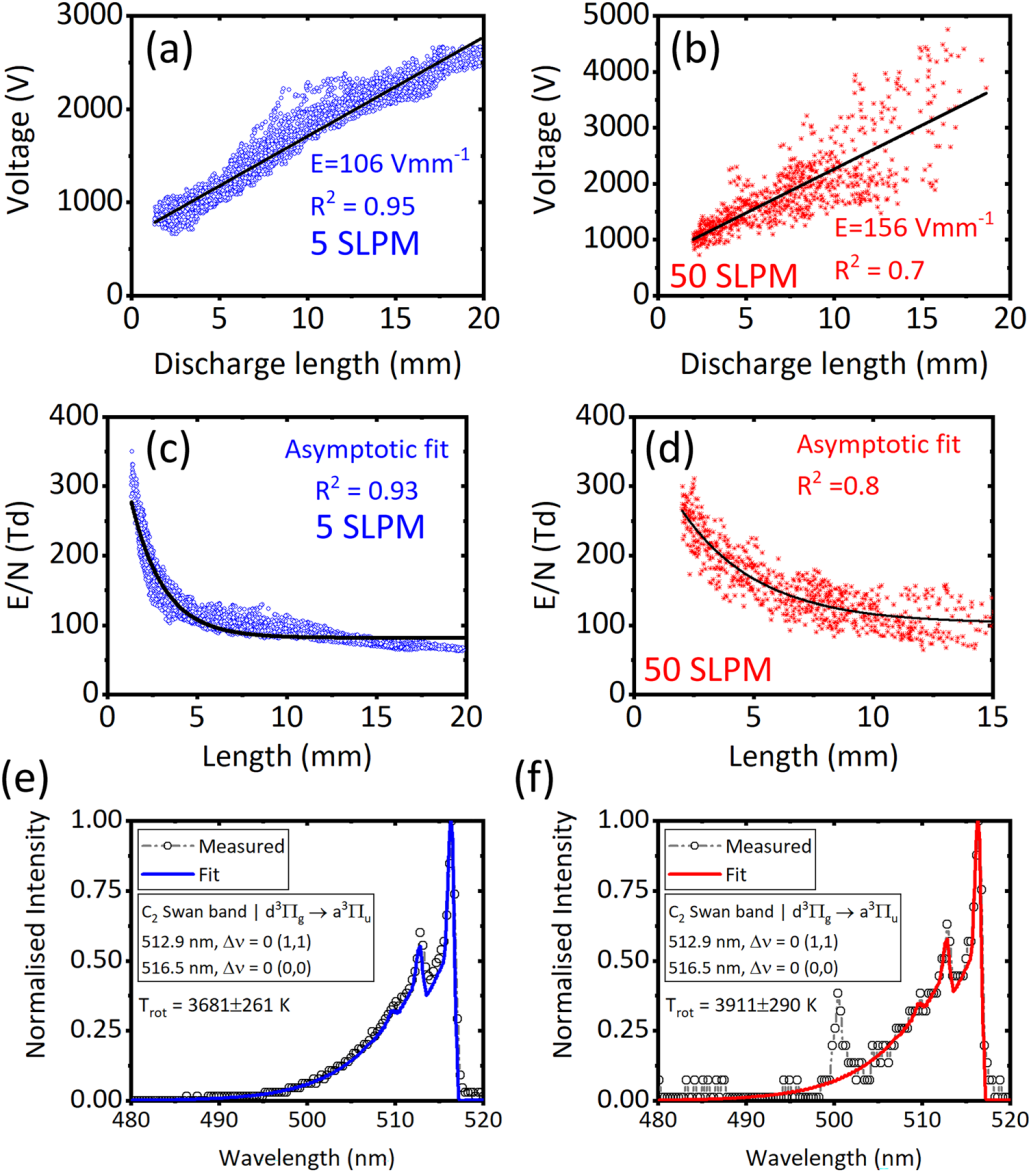
Root-mean-square of voltage as a function of discharge length and the linear fit for transitional (**a**) and turbulent (**b**) flow regimes. Reduced electric field as a function of discharge length, and the asymptotic fit, for transitional (**c**) and turbulent flow (**d**) regimes. The measured and fitted *OES* spectrum of $C_2$ Swan band for (**e**) transitional and (**f**) turbulent flow regimes.

The average *E* was calculated by fitting the linear function on *V* Vs. $l_d$ [see Fig. 5a,b]; this corresponds to the $\frac{E}{N}$ of 49–57 Td at transitional, and 77–90 Td at turbulent (approach-1), based on the average $T_{gas}$. However, alternatively, the variation of $\frac{E}{N}$ as a function of $l_d$ (or position of the discharge during its rotation) was investigated as shown in Fig. 5c,d (approach-2), using the same average $T_{gas}$. As can be seen, the $\frac{E}{N}$ in both the flow regimes were very high (100–300 Td) when the $l_d$ was between 3 and 5 mm, near $\delta$; this corresponds to a duration of 100 ms at 5 SLPM, and 1 ms at 50 SLPM based on the $f_{arc}$ of $\approx 11\,\hbox {Hz}$ and $167\pm 7\,\hbox {Hz}$, at 5 and 50 SLPM, respectively. As the discharge elongated during its rotation the $\frac{E}{N}$ approached asymptotic values of 82 Td at 5 SLPM, and $102\pm 3\,\hbox {Td}$ at 50 SLPM. Authors, have already observed the asymptotic trend of $\frac{E}{N}$ as a function of $l_d$ in their previous work in argon–*RGA*^18^, and attributed to the behaviour of *E* dropping self-consistently during the elongation of discharge, as expected in *GAD*^50^. The asymptotic $\frac{E}{N}$ characterized 94% (5 SLPM) and 92% (50 SLPM) of the rotational period, and for this reason, the asymptotic value was considered as realistic to use as input to chemical kinetics simulation, the approach the authors used in their previous work^18^. However, the correction can be made on the $\frac{E}{N}$ in the future, specifically at near $\delta$ positions, by obtaining spatio–temporal variation of $T_{gas}$ using ICCD cameras to further refine the model. The calculated $\frac{E}{N}$ based on both the approaches were in the typically acceptable range of 5–100 Td reported for gliding arcs^18,51^. Figure 5e,f show the measured and fitted *OES* spectrum of $\text {C}_2$ Swan band, having good agreement (matching of shape) between them, achieved using the optimization scheme provided in the SPECAIR tool. At 50 SLPM, an unidentified peak was found in the $\text {C}_2$ Swan band range [see Fig. 5f], which needs to be investigated in the future, and is less likely that it affected the estimated $T_{gas}$ using $\text {C}_2$ Swan band, which is the scope of this work. The rotation of the discharge at 5 and 50 SLPM captured using the *HSC* is shown in the Supplementary Videos titled “Video 1” and “Video 2”, respectively.

### Influence of flow regimes on plasma performance and chemistry

#### Methane conversion and energy efficiency

**Figure 6:**
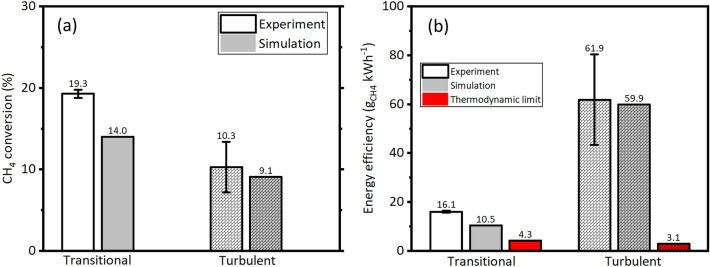
$\text {CH}_4$ conversion (**a**) and its energy efficiency, (**b**) at transitional, and turbulent flow regimes; estimated from experiment, simulation and thermodynamic equilibrium analysis.

Figure 6a shows the $\text {CH}_4$ conversion (experiment and simulation) at transitional and turbulent flow regimes. When the flow regime changed from transitional to turbulent flow due to increased flow rate (5 SLPM to 50 SLPM), the $\text {CH}_4$ conversion decreased by 46% (19.3% to 10.3%), similar to the observation reported by Zhang et al.^16^. The simulation of $\text {CH}_4$ conversion at transitional and turbulent flow regimes also predicted the decreasing trend, showing fairly good agreement with the experimental. Zhang et al.^16^ achieved $\text {CH}_4$ conversion of $\approx 15\%$ at $24\,\hbox {SLPM}$ (the maximum flow rate used in their work) by spending $\approx 0.3\,\hbox {ev}\cdot \hbox {molecule}^{-1}$; whereas in this work, by spending an order lesser energy of $\approx 0.03\,\hbox {eV}\cdot \hbox {molecule}^{-1}$, a $\text {CH}_4$ conversion of 10.3% was achieved at higher flow rate of 50 SLPM which is comparable with that of Zhang et al.^16^.

Figure 6b shows the $\eta _E$ at transitional and turbulent flow regimes. Experimental results showed that the $\eta _E$ enhanced by a factor of $\approx 3.9$, indicating the the $\text {CH}_4$ decomposition is energy efficient at turbulent flow (50 SLPM) than at the transitional flow (5 SLPM). Both the flow regimes showed higher $\eta _E$ than the thermodynamic equilibrium limit, by a factor of 3.7 (transitional) and 20 (turbulent), re-emphasizing the positive feature of the *NTP*, particularly *RGAs*. The $\eta _E$ calculated using the simulation results showed fairly good agreement with the experimental.

#### Dominant reactions in the consumption of methane

**Figure 7:**
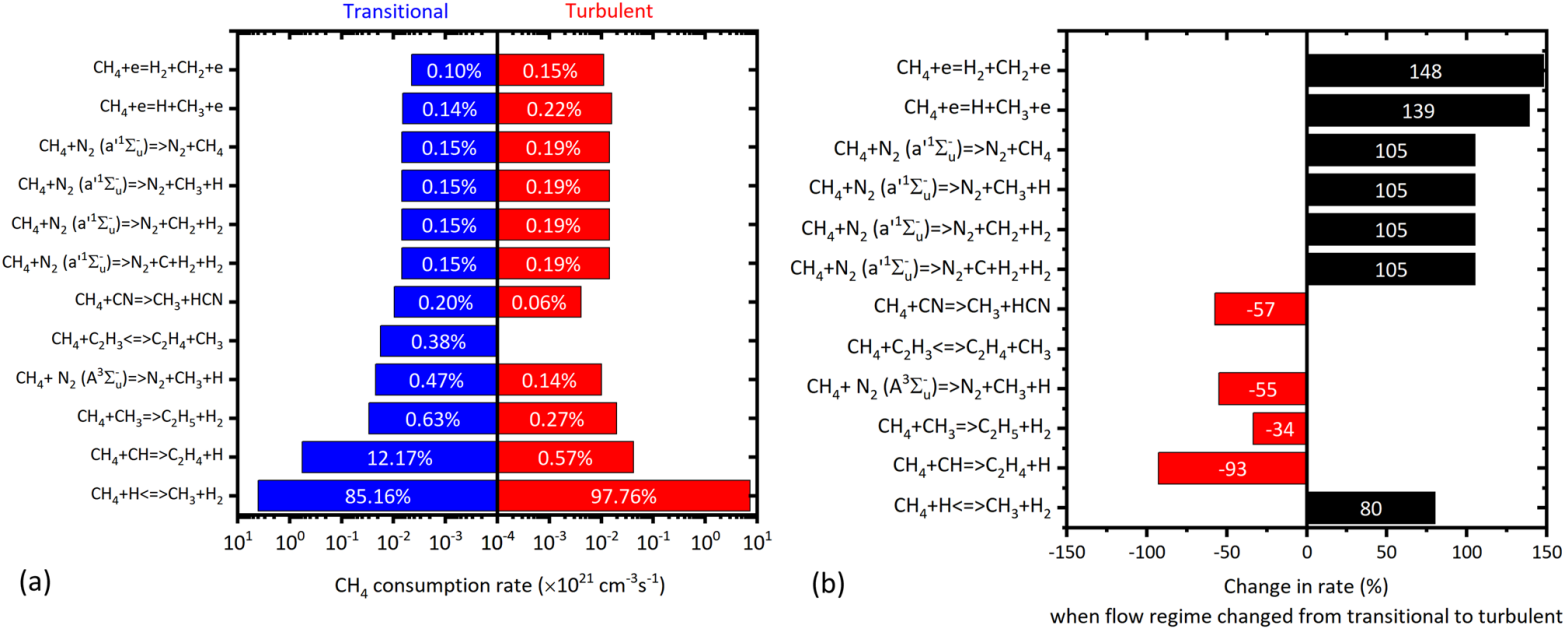
(**a**) Dominant reactions and their rates, with a relative contribution higher than 0.1% (shown as label) in the total consumption rate at transitional and turbulent flow regimes. (**b**) The % change in the rate of the dominant reactions due to flow regime change.

The $\text {CH}_4$ consumption rate was calculated as $4.72 \times 10^{21}\,\hbox {molecules}\cdot \hbox {cm}^{-3}\cdot \hbox {s}^{-1}$ at transitional flow, which increased to $7.4\times 10^{21}\,\hbox {molecules}\cdot \hbox {cm}^{-3}\cdot \hbox {s}^{-1}$ at turbulent flow. Figure 7a shows the dominant reactions contributing higher than 0.1% towards the total rate of the $\text {CH}_4$ consumption, at transitional and turbulent flow. At both the flow regimes, the first three dominant reactions was induced by the H, CH and $\text {CH}_3$ (referring as *critical species*), respectively as follows:

1. $$\text {CH}_4 + {\text {H}} <=> {\text {CH}}_3 + {\text {H}}_2$$ (favoured forward);
2. $$\text {CH}_4 + {\text {CH}} => {\text {C}}_2{\text {H}}_4 + {\text {H}}$$;
3. $$\text {CH}_4 + {\text {CH}}_3 => {\text {C}}_2{\text {H}}_5 + {\text {H}}_2$$.

The contribution of R1 was 85.16% at transitional flow, which increased to 97.76% at turbulent flow. Zhang et al.^46^ also reported the reaction induced by *H* atom/radical as the most dominant in their work. The R2 contributed 12.17% towards the total $\text {CH}_4$ consumption rate; however its contribution decreased to 0.57% ($< 1\%$) at turbulent flow. The R3 contributed $< 1\%$ in both the flow regimes, relatively higher at transitional. Though the first three dominant reactions were the same for both the flow regimes, the reactions that followed these three were different in transitional and turbulent. In transitional flow, the excited metastable triplet state of $\text {N}_2$ i.e. $\text {N}_2(A^3\Sigma _u^-)$, followed by the $\text {C}_2\text {H}_3$ were inducing the $4{{\text{th}}}$ and $5{\text{th}}$ dominant reactions, respectively [see Fig. 7a]; whereas in turbulent flow, direct-impact of electrons, followed by the metastable singlet state $\text {N}_2(a'^1\Sigma _u^-)$ induced the $4{{\text{th}}}$ and $5{{\text{th}}}$ dominant reactions, respectively [see Fig. 7a]. This indicated that the change in the flow regimes changed the dominant reactions contributing to the consumption of $\text {CH}_4$, despite their contribution being $< 1\%$. Zhang et al.^46^ reported that for operating flow rate of 6 SLPM in their *MRGA*, the second dominant reaction was initiated by the $C_2H_3$ contributing 2.4–4.8%; in this work, this reaction was observed to be only the $5{{\text{th}}}$ dominating reaction, contributing only 0.38 % at transitional flow (5 SLPM), and insignificant at turbulent flow. This indicated that the difference in the characteristics and operation inputs of the *RGAs* could likely affect the plasma chemistry—a preliminary evidence which has to be further investigated in the future.

Figure 7b shows the change in the rates of the dominant reactions shown in Fig. 7a. The rate of the direct electron-impact dissociation of $\text {CH}_4$ into $\text {H}_2$ and H was increased by 148% and 139%, respectively, due to the increased $\frac{E}{N}$ at turbulent flow. The rate of the reactions induced by the singlet $N_2(a'^1\Sigma _u^-)$ increased by $\approx$105%; followed by 80% increase in the rate of R1. When the flow regime changed from transitional to turbulent, the rates of reactions R2, R3, and reactions induced by $\text {N}_2(A^3\Sigma _u^-)$, and CN, decreased in the range of 34–93% [see Fig. 7b]. The increase/decrease in the process rates likely occurred due to the change in the $\frac{E}{N}$, and $T_{gas}$, the two major rate affecting factors—indicating the influence of flow regimes on plasma chemistry. the increase/decrease of $\text {CH}_4$ consumption reactions showed that the loss in the rate of consumption occurred due to few reactions were compensated by enhanced rate of few reactions, maintaining the net $\text {CH}_4$ consumption. Based on these observations, the drop of 46% in $\text {CH}_4$ was considered minimal, with the residence time shortened by almost an order, and the energy input by 60% at turbulent flow.

To gain further insights, the G–factor of the critical species such as $\text {H}, \text {CH}, \text {CH}_3, \text {N}_2(A^3\Sigma _u^-), \text {N}_2(a'^1\Sigma _u^-)$ that are involved in the dominant reactions (shown in Fig. 7) was investigated. The G-factor indicates the energy efficiency in generating a species through electron-impact reactions (*EIR*); the *EIR* corresponding to the critical species, and their G-factor are shown in Table 3. It is very clear that, a 200% rise in the G-factor of H in *EIR* : 1 likely increased the contribution of R1 to 97.76%, and R1’s rate by 80%, at turbulent flow. Similarly the case with $\text {N}_2(a'^1\Sigma _u^-)$ in *EIR* : 2, whose G-factor increased by 80%, promoting the rate of their $\text {CH}_4$ consumption reactions [see Fig. 7b]. The G-factor of CH from *EIR* : 4 dropped by 65% at turbulent flow, the likely reason for the contribution of R2 to drop seen earlier [see Fig. 7a]. The $\text {CH}_3$ was generated through both *EIR* : 1 and *EIR* : 4, which showed a rise of 200%, and a drop of 65% in the G-factor, respectively; however, the absolute G-factor values of *EIR* : 3 were larger than that of the *EIR* : 1, which could have only cascading effect to increase the concentration of $\text {CH}_3$. Though the G-factor of $\text {N}_2(A^3\Sigma _u^-)$ was increased by 61.19%, the contribution of this species in $\text {CH}_4$ consumption was dropped by 55% at turbulent flow. This is because, the reaction involving de-excitation of $\text {N}_2(A^3\Sigma _u^-)$ to $\text {N}_2$ by energy transfer with H was having higher rate than that of the reaction involving $\text {CH}_4$ consumption induced by $\text {N}_2(A^3\Sigma _u^-)$, an order higher at transitional, and the same order but larger in value at turbulent flow. Further, in both transitional and *turbulent* flow regime, the distribution of electron energy was larger for the process generating $\text {N}_2(a'^1\Sigma _u^-)$ (39% and *76%*) than for the process generating $\text {N}_2(A^3\Sigma _u^-)$ (4% and *6.6%*). The energy distribution in generating $\text {N}_2(a'^1\Sigma _u^-)$ was increased by $\approx$ 95%, corroborated by the 105% increase in the rate of the reactions induced by $\text {N}_2(a'^1\Sigma _u^-)$ in $\text {CH}_4$ consumption [see Fig. 7b]. The following reactions contributed to the formation of $\text {CH}_4$, yet their rates were 5–6 orders of magnitude lesser than the R1: $\text {C}_2\text {H}_5 + e => \text {CH}_4 + \text {CH} + e$; and $\text {CH}_3 + \text {C}_2\text {H}_5 = \text {C}_2\text {H}_4 + \text {CH}_4$.

**Table 3:** G-factor of critical species contributing further to $\text {CH}_4$ consumption reactions, generated from electron-impact reactions.

Electron-impact reactions (EIR)		Critical species generated	G-factor, molecules$\cdot$ (100 eV)^−1^		% change in G-factor
			Transitional	Turbulent	
EIR:1	$e + \text {CH}_4 => e + \text {CH}_3 + \text {H}$	$\text {CH}_3, \text {H}$	0.01	0.03	200
EIR:2	$e + \text {N}_2 => e + \text {N}_2(a'^1\Sigma _u^-)$	$\text {N}_2(a'^1\Sigma _u^-)$	0.05	0.09	80
EIR:3	$e + \text {N}_2 => e + \text {N}_2(A^3\Sigma _u^-)$	$\text {N}_2(A^3\Sigma _u^-)$	0.67	1.08	61.19
EIR:4	$e + \text {C}_2\text {H}_4 => e + \text {CH}_3 + \text {CH}$	$\text {CH}_3, \text {CH}$	0.20	0.07	− 65

#### Some comments on products of methane conversion, and application prospects of this RGA

Table 4 shows the volumetric composition of the selected products formed as a result of $\text {CH}_4$ conversion, and the Carbon balance. The products detected in the *GC* was $\text {H}_2$, $\text {C}_2\text {H}_2$, $\text {C}_2\text {H}_4$ and $\text {C}_2\text {H}_6$; hydrocarbons higher than $\text {C}_2$ were not detected by *GC*—consistent with the observations reported by Zhang et al.^16^. $\text {H}_2$ was the major product, and was an order higher than the others. The measured and the predicted $\text {H}_2$ showed fair agreement, indicating that the mechanism is also suitable to understand the underlying mechanisms of $\text {H}_2$ formation. The fair agreement between simulation and experiments results of $\text {CH}_4$ conversion, and $\text {H}_2$ composition, indicated that the mechanism is suitable for *RGA* of $T_{gas}$ $> 3000$ K, since the mechanism in this work was validated with the only available literature work having $T_{gas}$ of 1000–1500 K. The composition of the $\text {C}_2$ species were under-predicted by the simulation (see Table 4). The discrepancy is likely as the current chemistry did not consider the effect of mixing and temperature effects, that could promote chemistry outside the plasma zone, which has to be investigated in the future. The products’ composition were of ppmV level, and the detailed reactions mechanisms of the products are not presented in this paper. Further, the current work focused mainly on capturing the effect of flow regimes on decomposition of 1% of $\text {CH}_4$ in $\text {N}_2$–*RGA* as a scientific study.

**Table 4:** Composition of the products in the product gas, and carbon balance.

Flow regime (Flow rate)	Volumetric composition of selected species, ppmV				Carbon balance, %
	$H_2$		$\text {C}_2\text {H}_2$ + $\text {C}_2\text {H}_4$ + $\text {C}_2\text {H}_6$		
	Experiment	Simulation	Experiment	Simulation	
Transitional (5 SLPM)	$509\pm 16$	544	$82\pm 8^\text{a}$	$\approx 8$	$83 \pm 1$
Turbulent (50 SLPM)	$204.3\pm 41$	177	$\approx 50^\text{a}$	$\approx 0.1$	$91 \pm 3$

^a^Peaks of $\text{C}_2\text{H}_x$ were clearly detected/identified, and quantified based on the nearest calibration point i.e, 100 ppmV.

## Conclusions

In summary, this work has shown that the gas flow rate through the flow regime [(*Re*] influences the plasma characteristics such as $\frac{E}{N}$, $T_e$, $T_{gas}$, and the dominant chemical reactions involved in the decomposition of diluted hydrocarbon ($\text {CH}_4$) in nitrogen *RGA* reactor. Particularly, highly turbulent flow was indicating energy efficient conversion process. The detailed observations of this work are summarized as follows:

When the flow regime changed between transitional (5 SLPM) and turbulent (50 SLPM), the operation mode transitioned from glow to spark with an increase in average *E*, $T_e$ and $T_{gas}$, i.e., $106\rightarrow 156\,\hbox {V}\cdot \hbox {mm}^{-1}$, $1.62 \rightarrow 2.12\,\hbox {eV}$, and $3681 \rightarrow 3911\,\hbox {K}$, respectively. The $\eta _E$ increased by $\approx 3.9$ times ($16.1 \rightarrow 61.9\,\hbox {g}\cdot \hbox {kWh}^{-1}$), with values of both the regimes higher than that of the thermodynamic limit for the experimental conditions of this work at their corresponding $T_{gas}$. The conversion of $\text {CH}_4$ was dropped from 19.3 to 10.3%, which is likely due to an order reduction in the gas residence time due to increased flow rate. The simulation of the chemical kinetics for the operating conditions at transitional and turbulent flow regimes using the validated chemical mechanism revealed that the reactions induced by H, CH and $\text {CH}_3$ radicals were dominant in the consumption of $\text {CH}_4$ in both the flow regimes, yet differed in their contributions to the total $\text {CH}_4$ consumption rate between transitional and turbulent flow regime. Further, the rate of the $\text {CH}_4$ consumption reactions involving direct electron-impact, and singlet state of $\text {N}_2$ (metastable) were increased at turbulent flow by more than 100%. In contrast, rate of few dominant $\text {CH}_4$ reactions were decreased in the range of 34–93%. The G-factor of electron impact reactions generating the key species involved in the consumption of $\text {CH}_4$ were increased by more than 50%, the likely reason for energy efficient process at highly turbulent flow. These observations show evidence that the flow regimes by influencing the plasma characteristics/parameters, changes the plasma chemistry of $\text {CH}_4$ decomposition.

From the application point of view, based on this work, authors believe that the developed *RGA* reactor is suitable to decompose hydrocarbons of fugitive emissions (dilute concentrations), showing energy efficient operation at high flow rates—a promising feature for up-scaling. The conversion performance to be improved by optimising the *SEI*, $T_{gas}$ and $\frac{E}{N}$ using the reported chemical mechanism. The influence of additional gases such as $\text {O}_2$, $\text {CO}_2$ and moisture to be investigated in the future. The reactor can also be tuned for $\text {H}_2$ generation from $\text {CH}_4$/$\text {CO}_2$ decomposition, by optimizing the control parameters such as $\text {CH}_4/\text {CO}_2$ ratio to mimic natural gas or biogas; the corresponding chemical mechanism to optimise for maximum $\text {H}_2$ yield to be explored at that time.

The work highlighted the significance of flow regime (*Re*) which is often overlooked by the plasma community, and its effect should be investigated in other plasma sources like dielectric barrier discharge.

## Supplementary Information

Supplementary Information.

Supplementary Video 1.

Supplementary Video 2.

## Data Availability

The data that support the findings of this study are available from the corresponding author upon reasonable request.
