# Supplementary material for: Influence of flow regime on the decomposition of diluted methane in a nitrogen rotating gliding arc
Source: Sci Rep. 2022 Jul 9;12:11700. doi: 10.1038/s41598-022-14435-z (PMC9271092; doi:10.1038/s41598-022-14435-z)
Supplement: Supplementary file 1 — Supplementary Information. [file 41598_2022_14435_MOESM1_ESM.pdf]

Influence of flow regime on the decomposition of diluted methane in a nitrogen rotating gliding arc: supplementary material

Ananthanarasimhan J and Lakshminarayana Rao*

Centre for Sustainable Technologies, Indian Institute of Science, Bengaluru 560012, India

E-mail: `narayana@iisc.ac.in` (*corresponding author)

I. Description of the supplementary videos:

(1) Supplementary video titled "video-1": Rotation of the discharge (1% CH_4 in N_2) in this rotating gliding arc (RGA) at 5 SLPM or transitional flow regime, captured using the high–speed camera (HSC).

(2) Supplementary video titled "video-2": Rotation of the discharge (1% CH_4 in N_2) in this RGA at 50 SLPM or turbulent flow regime, captured using the HSC.

II. Processes considered in Chemical Workbench:

Table SI shows the list of reactions considered in the Chemical Workbench and the corresponding rate coefficient details.

Table SI: Processes considered and their rates for experimental conditions obtained using Chemical Workbench.

S. No.	Reaction	Rate coefficient: for electron–impact reaction	Rate coefficient: heavy particle or thermal reaction A (cm, molecules, s)	N	E_i (kJ/mole)	Ref.
1	$e + N_2 \Rightarrow e + N_2$	f(σ)				[1]
2	$e + N_2 \Rightarrow e + N_2(rot)$	f(σ)				[1]
3	$e + N_2 \Rightarrow e + N_2(V1)$	f(σ)				[1]
4	$e + N_2 \Rightarrow e + N_2(V2)$	f(σ)				[1]
5	$e + N_2 \Rightarrow e + N_2(V3)$	f(σ)				[1]
6	$e + N_2 \Rightarrow e + N_2(V4)$	f(σ)				[1]
7	$e + N_2 \Rightarrow e + N_2(V5)$	f(σ)				[1]
8	$e + N_2 \Rightarrow e + N_2(V6)$	f(σ)				[1]
9	$e + N_2 \Rightarrow e + N_2(V7)$	f(σ)				[1]
10	$e + N_2 \Rightarrow e + N_2(V8)$	f(σ)				[1]
11	$e + N_2 \Rightarrow e + N_2(A^3\Sigma)$	f(σ)				[2]
12	$e + N_2 \Rightarrow e + N_2(W^3\Delta)$	f(σ)				[1]
13	$e + N_2 \Rightarrow e + N_2(B^3\Pi)$	f(σ)				[1]
14	$e + N_2 \Rightarrow e + N_2(a^{'1}\Sigma)$	f(σ)				[1]
15	$e + N_2 \Rightarrow e + N_2(a^1\Pi)$	f(σ)				[1]
16	$e + N_2 \Rightarrow e + N_2(W^1\Delta)$	f(σ)				[1]
17	$e + N_2 \Rightarrow e + N + N$	f(σ)				[3]
18	$e + N_2 \Rightarrow e + N_2(E^3\Sigma)$	f(σ)				[1]
19	$e + N_2 \Rightarrow e + N_2(C^3\Pi)$	f(σ)				[1]
20	$e + N_2 \Rightarrow e + N_2(a"\Sigma)$	f(σ)				[1]
21	$e + N_2 \Rightarrow e + N_2(SUM)$	f(σ)				[1]
22	$e + N_2 \Rightarrow e + e + N_2^+$	f(σ)				[1]
23	$e + N_2 \Rightarrow e + e + N_2^+(B^2\Sigma)$	f(σ)				[1]

Continued on next page

Table SI – continued from previous page

S. No.	Reaction	Rate coefficient				Ref.
		electron–impact reaction	heavy particle or thermal reaction			
			A (cm, molecules, s)	N	E_i (kJ/mole)	
24	$e + CH_4 \Rightarrow e + CH_4$	f(σ)				[4]
25	$e + CH_4 \Rightarrow e + e + CH_4^+$	f(σ)				[4]
26	$e + CH_4 \Rightarrow e + e + H + CH_3^+$	f(σ)				[4]
27	$e + CH_3 \Rightarrow e + CH_3$	f(σ)				[5]
28	$e + CH_3 \Rightarrow e + 2H + CH$	f(σ)				[6, 7]
30	$e + CH_3 \Rightarrow e + CH_3^+ + e$	f(σ)				[5]
31	$e + CH_3 \Rightarrow e + e + H + CH_2^+$	f(σ)				[5]
32	$e + CH_2 \Rightarrow e + CH_2$	f(σ)				[5]
33	$e + CH_2 \Rightarrow e + H + CH$	f(σ)				[6, 7]
34	$e + CH_2 \Rightarrow e + H_2 + C$	f(σ)				[6, 7]
35	$e + CH_2 \Rightarrow e + 2H + C$	f(σ)				[6, 7]
36	$e + CH_2 \Rightarrow e + e + CH_2^+$	f(σ)				[5]
37	$e + CH_2 \Rightarrow e + e + CH^+ + H$	f(σ)				[5]
38	$e + CH \Rightarrow e + CH$	f(σ)				[5]
39	$e + CH \Rightarrow e + H + C$	f(σ)				[6, 7]
40	$e + CH \Rightarrow e + e + CH^+$	f(σ)				[5]
41	$e + CH \Rightarrow e + e + H + C^+$	f(σ)				[5]
42	$e + H_2 \Rightarrow e + H_2$	f(σ)				[8]
43	$e + H_2 \Rightarrow e + H + H$	f(σ)				[9]
44	$e + H_2 \Rightarrow e + e + H_2^+$	f(σ)				[10]
45	$e + C_2H_2 \Rightarrow e + C_2H_2$	f(σ)				[5]
46	$e + C_2H_2 \Rightarrow e + C_2H_2(v5)$	f(σ)				[5]
47	$e + C_2H_2 \Rightarrow e + C_2H_2(v2)$	f(σ)				[5]
48	$e + C_2H_2 \Rightarrow e + C_2H_2(v31)$	f(σ)				[5]
49	$e + C_2H_2 \Rightarrow e + C_2H_2(*)$	f(σ)				[5]

Continued on next page

Table SI – continued from previous page

S. No.	Reaction	Rate coefficient				Ref.
		electron–impact reaction	heavy particle or thermal reaction			
			A (cm, molecules, s)	N	E_i (kJ/mole)	
50	$e + C_2H_2 \Rightarrow e + C_2H_2(*)$	f(σ)				[5]
51	$e + C_2H_2 \Rightarrow e + C_2H_2(*)$	f(σ)				[5]
52	$e + C_2H_2 \Rightarrow e + C_2H + H$	f(σ)				[11]
54	$e + C_2H_2 \Rightarrow e + 2H + C_2$	f(σ)				[11]
55	$e + C_2H_2 \Rightarrow e + CH + CH$	f(σ)				[11]
56	$e + C_2H_2 \Rightarrow e + CH_2 + C$	f(σ)				[11]
57	$e + C_2H \Rightarrow e + H + C_2$	f(σ)				[11]
58	$e + C_2H \Rightarrow e + CH + C$	f(σ)				[11]
59	$e + C_2H_3 \Rightarrow e + C_2H_2 + H$	f(σ)				[11]
60	$e + C_2H_3 \Rightarrow e + C_2H + H_2$	f(σ)				[11]
61	$e + C_2H_3 \Rightarrow e + C_2H + 2H$	f(σ)				[11]
62	$e + C_2H_3 \Rightarrow e + C_2 + H_2 + H$	f(σ)				[11]
63	$e + C_2H_3 \Rightarrow e + CH_2 + CH$	f(σ)				[11]
64	$e + C_2H_3 \Rightarrow e + CH_3 + C$	f(σ)				[11]
65	$e + C_2H_4 \Rightarrow e + C_2H_3 + H$	f(σ)				[11]
66	$e + C_2H_4 \Rightarrow e + C_2H_2 + H_2$	f(σ)				[11]
67	$e + C_2H_4 \Rightarrow e + C_2H_2 + 2H$	f(σ)				[11]
68	$e + C_2H_4 \Rightarrow e + C_2H + H + H_2$	f(σ)				[11]
69	$e + C_2H_4 \Rightarrow e + CH_3 + CH$	f(σ)				[11]
70	$e + C_2H_4 \Rightarrow e + CH_2 + CH_2$	f(σ)				[11]
71	$e + C_2H_4 \Rightarrow e + CH_4 + C$	f(σ)				[11]
72	$e + C_2H_5 \Rightarrow e + C_2H_4 + H$	f(σ)				[11]
73	$e + C_2H_5 \Rightarrow e + H_2 + C_2H_3$	f(σ)				[11]
74	$e + C_2H_5 \Rightarrow e + 2H + C_2H_3$	f(σ)				[11]
75	$e + C_2H_5 \Rightarrow e + H + H_2 + C_2H_2$	f(σ)				[11]

Continued on next page

Table SI – continued from previous page

S. No.	Reaction	Rate coefficient				Ref.
		electron–impact reaction	heavy particle or thermal reaction			
			A (cm, molecules, s)	N	E_i (kJ/mole)	
76	$e + C_2H_5 \Rightarrow e + 2H_2 + C_2H$	f(σ)				[11]
77	$e + C_2H_5 \Rightarrow e + CH_4 + CH$	f(σ)				[11]
78	$e + C_2H_5 \Rightarrow e + CH_2 + CH_3$	f(σ)				[11]
79	$e + C_2H_6 \Rightarrow e + C_2H_5 + H$	f(σ)				[11]
80	$e + C_2H_6 \Rightarrow e + C_2H_4 + H_2$	f(σ)				[11]
81	$e + C_2H_6 \Rightarrow e + C_2H_3 + H + H_2$	f(σ)				[11]
82	$e + C_2H_6 \Rightarrow e + 2H_2 + C_2H_2$	f(σ)				[11]
83	$e + C_2H_6 \Rightarrow e + CH_2 + CH_4$	f(σ)				[11]
84	$e + C_2H_6 \Rightarrow e + CH_3 + CH_3$	f(σ)				[11]
85	$CH_4 + N_2(A^3\Sigma) \Rightarrow N_2 + CH_3 + H$		1.50E-12	0	0	[12–14]
86	$CH_4 + N_2(A^3\Sigma) \Rightarrow N_2 + CH_2 + H_2$		1.35E-13	0	0	[13, 14]
87	$CH_4 + N_2(A^3\Sigma) \Rightarrow N_2 + CH_4$		3.20E-15	0	0	[13, 15]
88	$CH_3 + N_2(A^3\Sigma) \Rightarrow N_2 + CH_2 + H$		1.00E-13	0	0	[13, 14]
89	$H_2 + N_2(A^3\Sigma) \Rightarrow N_2 + H + H$		2.40E-15	0	0	[13–15]
90	$H + N_2(A^3\Sigma) \Rightarrow N_2 + H$		2.10E-10	0	0	[15]
91	$C_2H_4 + N_2(A^3\Sigma) \Rightarrow N_2 + C_2H_2 + H_2$		1.10E-10	0	0	[14]
92	$C_3H_4 + N_2(A^3\Sigma) \Rightarrow N_2 + CH_2 + C_2H_2$		2.80E-10	0	0	[13, 14]
93	$HCN + N_2(A^3\Sigma) \Rightarrow N_2 + CN + H$		6.00E-12	0	0	[13, 14]
94	$CH_4 + N_2(a^{'1}\Sigma) \Rightarrow N_2 + C + H_2 + H_2$		3.00E-10	0	0	[12–14]
95	$CH_4 + N_2(a^{'1}\Sigma) \Rightarrow N_2 + CH_2 + H_2$		3.00E-10	0	0	[14]
96	$CH_4 + N_2(a^{'1}\Sigma) \Rightarrow N_2 + CH_3 + H$		3.00E-10	0	0	[12]
97	$CH_4 + N_2(a^{'1}\Sigma) \Rightarrow N_2 + CH_4$		3.00E-10	0	0	[13]
98	$H_2 + N_2(a^{'1}\Sigma) \Rightarrow N_2 + H + H$		2.60E-11	0	0	[12–14]
99	$H_2 + N_2(a^{'1}\Sigma) \Rightarrow N_2 + H_2$		2.10E-10	0	0	[15]
100	$H + N_2(a^{'1}\Sigma) \Rightarrow N_2 + H$		2.10E-10	0	0	[15]

Continued on next page

Table SI – continued from previous page

S. No.	Reaction	Rate coefficient				Ref.
		electron–impact reaction	heavy particle or thermal reaction			
			A (cm, molecules, s)	N	E_i (kJ/mole)	
101	$C_2H_2 + N_2(a^{'1}\Sigma) \Rightarrow N_2 + H + C_2H$		3.00E-10	0	0	[12]
102	$C_2H_4 + N_2(a^{'1}\Sigma) \Rightarrow N_2 + H_2 + C_2H_2$		2.00E-10	0	0	[12]
103	$C_2H_4 + N_2(a^{'1}\Sigma) \Rightarrow N_2 + H + C_2H_3$		2.00E-10	0	0	[12]
104	$CH_4 \Rightarrow CH_3 + H$		7.51E-07	0	380	[16]
105	$CH_3 \Rightarrow CH_2 + H$		1.69E-08	0	379	[16]
106	$CH_3 \Rightarrow H_2 + CH$		1.66E-08	0	357	[16]
107	$CH_2 \Rightarrow C + H_2$		2.66E-10	0	268	[16]
108	$CH_2 \Rightarrow CH + H$		9.33E-09	0	375	[16]
109	$CH \Rightarrow C + H$		3.16E-10	0	280	[16]
110	$H + H + M \Leftrightarrow H_2 + M$ $H_2/0.00/\,CH_4/2.00/\,C_2H_6/3.00/$ [a]		1.00E-30	0	0	[17]
111	$2H + H_2 \Leftrightarrow 2H_2$		2.48E-30	-0.6	0	[18]
112	$H_2 + H \Rightarrow H + H + H$		0.00014	-1	457.3	[16]
113	$CH_4 + CH_3 \Rightarrow C_2H_5 + H_2$		1.70E-11	0	95.6	[19]
114	$CH_4 + CH_2 \Rightarrow CH_3 + CH_3$		7.10E-12	0	42	[19]
115	$CH_4 + CH \Rightarrow C_2H_4 + H$		9.74E-11	0	0	[12]
116	$CH_4 + C_2H_5 \Leftrightarrow C_2H_6 + CH_3$		1.43E-25	4.1	52.5	[16]
117	$CH_4 + C_2H_3 \Leftrightarrow C_2H_4 + CH_3$		2.41E-24	4	22.9	[16]
118	$CH_4 + C_2H \Leftrightarrow C_2H_2 + CH_3$		3.01E-12	0	2.1	[12]
119	$CH_4 + H \Leftrightarrow CH_3 + H_2$		1.10E-15	1.62	45.3	[20–22]
120	$CH_3 + CH_3 \Rightarrow C_2H_5 + H$		8.26E-12	0.1	44.4	[16]
121	$CH_3 + CH_3 \Rightarrow C_2H_6$		5.42E-08	-1.1	1330	[16]
122	$CH_3 + CH_3 \Rightarrow C_2H_4 + H_2$		1.66E-08	0	134	[16]
123	$CH_3 + C_2H_5 \Rightarrow C_2H_4 + CH_4$		3.25E-11	-0.5	0	[16]
124	$CH_3 + CH_2 \Rightarrow C_2H_4 + H$		7.01E-11	0	0	[12]

Continued on next page

Table SI – continued from previous page

S. No.	Reaction	Rate coefficient				Ref.
		electron–impact reaction	heavy particle or thermal reaction			
			A (cm, molecules, s)	N	E_i (kJ/mole)	
125	$CH_3 + CH \Rightarrow C_2H_3 + H$		5.00E-11	0	0	[19]
126	$CH_3 + C_2H_3 \Rightarrow C_2H_2 + CH_4$		6.51E-13	0	0	[12]
127	$CH_3 + C \Rightarrow C_2H_2 + H$		8.30E-11	0	0	[23]
128	$CH_3 + H \Leftrightarrow CH_2 + H_2$		1.00E-10	0	63.2	[16]
129	$CH_2 + CH \Rightarrow C_2H_2 + H$		6.60E-11	0	0	[23]
130	$CH_2 + C \Rightarrow C_2H + H$		8.30E-11	0	0	[19]
131	$CH_2 + CH_2 \Rightarrow C_2H_2 + H_2$		2.62E-09	0	50	[16]
132	$CH_2 + CH_2 \Rightarrow C_2H_2 + H + H$		3.32E-10	0	46	[16]
133	$CH_2 + CH_2 \Rightarrow C_2H_4$		1.70E-12	0	0	[23]
134	$CH_2 + C_2H_3 \Rightarrow C_2H_2 + CH_3$		3.01E-11	0	0	[16]
135	$CH_2 + C_2H_5 \Rightarrow C_2H_4 + CH_3$		3.01E-11	0	0	[16]
136	$CH_2 + C_2H \Rightarrow C_2H_2 + CH$		3.00E-11	0	0	[24]
137	$CH_2 + H \Leftrightarrow CH + H_2$		1.00E-11	0	-7.5	[16]
138	$CH + CH \Rightarrow C_2H_2$		2.00E-10	0	0	[23]
139	$CH + H \Rightarrow C + H_2$		1.31E-10	0	0.7	[16]
140	$C_2 + M \Rightarrow C + C + M$		6.18E-10	0	580.5	[25]
141	$C + C \Leftrightarrow C_2$		2.16E-11	0	0	[13]
142	$H_2 + C_2 \Rightarrow C_2H_2$		1.77E-10	0	12.2	[13]
143	$H_2 + C_2 \Rightarrow C_2H + H$		1.77E-10	0	12.2	[13]
144	$CH + M \Rightarrow C + H + M$		3.15E-10	0	280.2	[25]
145	$CH_2 + M \Rightarrow H_2 + C + M$		2.16E-10	0	246.9	[25]
146	$C_2H_6 + C_2H_3 \Rightarrow C_2H_5 + C_2H_4$		9.90E-22	3.3	43.9	[24]
147	$CH_4 + C_2 \Rightarrow C_2H + CH_3$		5.50E-11	0	2.5	[13]
148	$C_2H_6 + H \Leftrightarrow C_2H_5 + H_2$		2.39E-15	1.5	31	[16]
149	$C_2H_6 + C_2H \Rightarrow C_2H_2 + C_2H_5$		5.99E-12	0	0	[16]

Continued on next page

Table SI – continued from previous page

S. No.	Reaction	Rate coefficient				Ref.
		electron–impact reaction	heavy particle or thermal reaction			
			A (cm, molecules, s)	N	E_i (kJ/mole)	
150	$C_2H_5 + C_2H_5 \Rightarrow C_2H_6 + C_2H_4$		2.41E-12	0	0	[16]
151	$C_2H_5 + C_2H_3 \Rightarrow C_2H_6 + C_2H_2$		1.86E-11	0.5	0	[13]
152	$C_2H_5 + C_2H \Rightarrow C_2H_4 + C_2H_2$		3.01E-12	0	0	[16]
153	$C_2H_5 + C_2H_3 \Rightarrow C_2H_4 + C_2H_4$		4.98E-12	0	0	[26, 27]
154	$C_2H_5 + H \Rightarrow C_2H_4 + H_2$		3.01E-12	0	0	[16]
155	$C_2H_5 + H \Rightarrow C_2H_6$		9.04E-11	0.16	0	[16]
156	$C_2H_4 + C_2H \Rightarrow C_2H_2 + C_2H_3$		1.40E-10	0	0	[12]
157	$C_2H_4 + H \Leftrightarrow C_2H_3 + H_2$		9.00E-10	0	62.3	[28]
158	$C_2H_3 + C_2H_3 \Rightarrow C_2H_4 + C_2H_2$		1.60E-12	0	0	[16]
159	$C_2H_3 + C_2H \Rightarrow C_2H_2 + C_2H_2$		1.60E-12	0	0	[16]
160	$C_2H_3 + H \Rightarrow C_2H_2 + H_2$		2.01E-11	0	0	[16]
161	$C_2H_2 + H \Leftrightarrow C_2H + H_2$		1.00E-10	0	93.1	[16]
162	$C_2H_2 + H \Rightarrow C_2H_3$		1.10E-23	3.7	39.7	[18]
163	$CH + C_2H_2 \Rightarrow C_3H_2 + H$		1.83E-11	0	0	[29]
164	$C_2H + C_2H \Rightarrow C_2H_2 + C_2$		3.00E-12	0	0	[24]
165	$CH_4 + C_3H_7 \Leftrightarrow C_3H_8 + CH_3$		4.01E-26	4	45.5	[16]
166	$CH_3 + C_2H_5 \Rightarrow C_3H_8$		5.60E-11	0	0	[16]
167	$CH_3 + C_3H_7 \Rightarrow C_3H_6 + CH_4$		3.90E-11	-0.3	0	[16]
168	$CH_3 + C_3H_6 \Rightarrow C_3H_5 + CH_4$		1.24E-19	0	0	[12]
169	$CH_2 + C_2H_6 \Rightarrow C_3H_8$		5.60E-11	0	0	[30]
170	$CH_2 + C_3H_8 \Rightarrow C_3H_7 + CH_3$		1.50E-24	3.6	29.9	[16]
171	$CH_2 + C_3H_7 \Rightarrow C_2H_4 + C_2H_5$		3.01E-11	0	0	[12]
172	$CH_2 + C_3H_7 \Rightarrow C_3H_6 + CH_3$		3.01E-11	0	0	[12]
173	$CH_2 + C_3H_6 \Rightarrow C_3H_5 + CH_3$		1.20E-12	0	25.9	[16]
174	$C + C_2H_2 \Rightarrow C_3H_2$		5.95E-10	0	0	[13]

Continued on next page

Table SI – continued from previous page

S. No.	Reaction	Rate coefficient				Ref.
		electron–impact reaction	heavy particle or thermal reaction			
			A (cm, molecules, s)	N	E_i (kJ/mole)	
175	$C_2H_6 + C_3H_7 \Leftrightarrow C_3H_8 + C_2H_5$		4.21E-25	3.8	37.8	[16]
176	$C_2H_5 + C_3H_7 \Rightarrow C_3H_8 + C_2H_4$		1.91E-12	0	0	[12]
177	$C_2H_5 + C_3H_7 \Rightarrow C_3H_6 + C_2H_6$		2.41E-12	0	0	[16]
178	$C_2H_5 + C_3H_6 \Rightarrow C_3H_5 + C_2H_6$		3.70E-24	3.5	27.8	[16]
179	$C_2H_5 + C_3H_5 \Leftrightarrow C_3H_6 + C_2H_4$		4.30E-12	0	-0.6	[16]
180	$C_2H_3 + C_3H_8 \Rightarrow C_2H_4 + C_3H_7$		9.99E-22	3.3	43.9	[16]
181	$C_2H_3 + C_3H_7 \Rightarrow C_2H_2 + C_3H_8$		2.01E-12	0	0	[12]
182	$C_2H_3 + C_3H_7 \Rightarrow C_2H_4 + C_3H_6$		2.01E-12	0	0	[12]
183	$C_2H_3 + C_3H_6 \Rightarrow C_2H_4 + C_3H_5$		6.58E-19	0	0	[12]
184	$C_2H_3 + C_3H_5 \Rightarrow C_2H_2 + C_3H_6$		8.00E-12	0	0	[12]
185	$C_2H + C_3H_8 \Rightarrow C_2H_2 + C_3H_7$		5.99E-12	0	0	[12]
186	$C_2H + C_3H_7 \Rightarrow C_3H_6 + C_2H_2$		1.00E-11	0	0	[16]
187	$C_2H + C_3H_6 \Rightarrow C_3H_5 + C_2H_2$		5.99E-12	0	0	[12]
188	$C_3H_8 + H \Leftrightarrow C_3H_7 + H_2$		2.20E-18	2.5	28.3	[16]
189	$C_3H_7 + C_3H_7 \Rightarrow C_3H_6 + C_3H_8$		2.81E-12	0	0	[12]
190	$C_3H_7 + C_3H_6 \Rightarrow C_3H_5 + C_3H_8$		3.70E-24	3.5	27.8	[16]
191	$C_3H_7 + C_3H_5 \Rightarrow C_3H_6 + C_3H_6$		2.41E-12	0	-0.6	[16]
192	$C_3H_7 + H \Rightarrow C_3H_6 + H_2$		3.01E-12	0	0	[12]
193	$C_3H_6 + H \Rightarrow C_3H_5 + H_2$		6.94E-15	0	0	[12]
194	$CH_4 + CN \Rightarrow CH_3 + HCN$		1.50E-19	2.6	-1.2	[16]
195	$CH_4 + N \Rightarrow CH_3 + NH$		1.04E-16	0	0	[23]
196	$CH_3 + N \Rightarrow H_2 + HCN$		1.40E-11	0	0	[12]
197	$CH_2 + NH \Rightarrow 2H + HCN$		5.00E-11	0	0	[23]
198	$CH + NH \Rightarrow H + HCN$		8.30E-11	0	0	[23]
199	$CH + NH_2 \Rightarrow 2H + HCN$		5.00E-11	0	0	[23]

Continued on next page

Table SI – continued from previous page

S. No.	Reaction	Rate coefficient				Ref.
		electron–impact reaction	heavy particle or thermal reaction			
			A (cm, molecules, s)	N	E_i (kJ/mole)	
200	$CH_2 + N \Rightarrow H + HCN$		5.00E-11	0	2.1	[12]
201	$CH_2 + N \Rightarrow H + H + CN$		1.60E-11	0	0	[12]
202	$CH_2 + N \Rightarrow H_2 + CN$		1.60E-11	0	0	[12]
203	$C + N_2 \Rightarrow CN + N$		1.04E-10	0	191.2	[12]
204	$C_2H_6 + CN \Rightarrow HCN + C_2H_5$		2.00E-19	2.8	-7.5	[16]
205	$C_2H_4 + N \Rightarrow HCN + CH_3$		3.30E-14	0	2.9	[12]
206	$C_2H_2 + N \Rightarrow HCN + CH$		2.70E-15	0	0	[12]
207	$C_3H_8 + CN \Rightarrow C_3H_7 + HCN$		6.76E-12	0	0	[13]
208	$C_3H_6 + N \Rightarrow HCN + C_2H_5$		1.94E-13	0	5.4	[12]
209	$H_2 + N \Leftrightarrow NH + H$		1.69E-09	0	150.5	[12]
210	$H_2 + CN \Rightarrow HCN + H$		4.90E-19	2.5	39.3	[16]
211	$H + NH_2 \Rightarrow NH + H_2$		1.00E-11	0	0	[12]
212	$H + NH_2 + M \Rightarrow NH_3 + M$		6.00E-30	0	0	[12]
213	$N_2 + CN \Rightarrow N_2 + C + N$		4.15E-10	0	586.5	[12]
214	$N + CH \Rightarrow CN + H$		2.10E-11	0	0	[12]
215	$N + CN \Rightarrow C + N_2$		3.01E-10	0	0	[16]
216	$NH_3 + H \Rightarrow H_2 + NH_2$		9.46E-20	2.8	42.7	[12]
217	$NH_2 + N \Rightarrow N_2 + H + H$		1.20E-10	0	0	[12]
218	$NH_2 + NH_2 \Rightarrow NH + NH_3$		8.30E-11	0	41.8	[12]
219	$NH + N \Rightarrow H + N_2$		8.60E-12	0.5	0	[13]
220	$NH + NH + M \Rightarrow H_2 + N_2 + M$		1.00E-33	0	0	[12]
221	$NH + NH \Rightarrow NH_2 + N$		9.40E-25	3.9	1.4	[16]
222	$NH + NH \Rightarrow N_2 + H + H$		1.20E-09	0	0	[12]
223	$NH + NH \Rightarrow N_2 + H_2$		1.70E-11	0	0	[12]
224	$N + H \Rightarrow NH$		5.02E-32	0	0	[12]

Continued on next page

Table SI – continued from previous page

S. No.	Reaction	Rate coefficient: electron–impact reaction	Rate coefficient: heavy particle or thermal reaction — A (cm, molecules, s)	N	E_i (kJ/mole)	Ref.
225	$e + C_2H_2 \Rightarrow e + e + C_2H_2^+$	f(σ)				[5]
226	$e + CH_3 \Rightarrow e + H + CH_2$	f(σ)				[6, 7]
227	$e + CH_3 \Rightarrow e + CH + H_2$	f(σ)				[6, 7]
228	$N_2(rot) + M \Rightarrow N_2 + M$		1.00E-09	0	0	[b]
229	$N_2(V1) + M \Rightarrow N_2 + M$		1.00E-09	0	0	[b]
230	$N_2(V2) + M \Rightarrow N_2 + M$		1.00E-09	0	0	[b]
231	$N_2(V3) + M \Rightarrow N_2 + M$		1.00E-09	0	0	[b]
232	$N_2(V5) + M \Rightarrow N_2 + M$		1.00E-06	0	0	[b]
233	$N_2(V4) + M \Rightarrow N_2 + M$		1.00E-09	0	0	[b]
234	$N_2(V6) + M \Rightarrow N_2 + M$		1.00E-09	0	0	[b]
235	$N_2(V8) + M \Rightarrow N_2 + M$		1.00E-06	0	0	[b]
236	$N_2(V7) + M \Rightarrow N_2 + M$		1.00E-09	0	0	[b]
237	$N_2(SUM) + M \Rightarrow N_2 + M$		1.00E-09	0	0	[b]
238	$e + CH_4 \Rightarrow e + H + CH_3$	f(σ)				[6, 7]
239	$e + CH_4 \Rightarrow e + H_2 + CH_2$	f(σ)				[6, 7]
240	$e + CH_4 \Rightarrow e + H + H_2 + CH$	f(σ)				[6, 7]
241	$e + CH_4 \Rightarrow e + C + 2H_2$	f(σ)				[6, 7]
242	$e + CH_4 \Rightarrow e + CH_4(v24)$	f(σ)				[4]
243	$e + CH_4 \Rightarrow e + CH_4(v13)$	f(σ)				[4]
244	$e + CH_4 \Rightarrow e + CH_4(*)$	f(σ)				[4]
245	$e + CH_4 \Rightarrow e + CH_4(*)$	f(σ)				[4]
246	$e + CH_4 \Rightarrow e + CH_4(*)$	f(σ)				[4]
247	$e + CH_4 \Rightarrow e + CH_4(*)$	f(σ)				[4]
248	$C_2H_2(v5) + M \Rightarrow C_2H_2 + M$		1.00E-09	0	0	[b]
249	$C_2H_2(v2) + M \Rightarrow C_2H_2 + M$		1.00E-09	0	0	[b]

Continued on next page

Table SI – continued from previous page

S. No.	Reaction	Rate coefficient: electron–impact reaction	Rate coefficient: heavy particle or thermal reaction: A (cm, molecules, s)	N	E_i (kJ/mole)	Ref.
250	$C_2H_2(v31) + M \Rightarrow C_2H_2\ + M$		1.00E-09	0	0	[b]
251	$C_2H_2(*) + M \Rightarrow C_2H_2\ + M$		1.00E-09	0	0	[b]
252	$CH_4(v24) + M \Rightarrow M + CH_4$ $CH_4/0.00$ [a]		0.001	0	0	[b]
253	$CH_4(v13) + M \Rightarrow M + CH_4$ $CH_4/0.00$ [a]		0.001	0	0	[b]
254	$CH_4(*) + M \Rightarrow M + CH_4$ $CH_4/0.00$ [a]		0.001	0	0	[b]
255	$e + H_2 \Rightarrow e + H_2(VIB)$	f(σ)				[31]
256	$e + H_2 \Rightarrow e + H_2(b^3\Sigma)$	f(σ)				[10]
257	$e + H_2 \Rightarrow e + H_2(*11.4)$	f(σ)				[10]
258	$e + H_2 \Rightarrow e + H_2(*11.75)$	f(σ)				[10]
259	$e + H_2 \Rightarrow e + H_2(*11.8)$	f(σ)				[10]
260	$e + H_2 \Rightarrow e + H_2(*12.4)$	f(σ)				[10]
261	$e + H_2 \Rightarrow e + H_2(*13.4)$	f(σ)				[10]
262	$e + H_2 \Rightarrow e + H_2(*13.8)$	f(σ)				[10]
263	$e + H_2 \Rightarrow e + H_2(*14)$	f(σ)				[10]
264	$e + H_2 \Rightarrow e + H_2(*14.6)$	f(σ)				[10]
265	$H_2(VIB) + M \Rightarrow H_2\ + M$		0.001	0	0	[b]
266	$H_2(b^3\Sigma) + M \Rightarrow H_2\ + M$		0.001	0	0	[b]
267	$H_2(*11.4) + M \Rightarrow H_2\ + M$		0.001	0	0	[b]
268	$H_2(*11.75) + M \Rightarrow H_2\ + M$		0.001	0	0	[b]
269	$H_2(*11.8) + M \Rightarrow H_2\ + M$		0.001	0	0	[b]
270	$H_2(*12.4) + M \Rightarrow H_2\ + M$		0.001	0	0	[b]
271	$H_2(*13.4) + M \Rightarrow H_2\ + M$		0.001	0	0	[b]

Continued on next page

Table SI – continued from previous page

S. No.	Reaction	Rate coefficient				Ref.
		electron–impact reaction	heavy particle or thermal reaction			
			A (cm, molecules, s)	N	E_i (kJ/mole)	
272	$H_2(*13.8) + M \Rightarrow H_2 + M$		0.001	0	0	[b]
273	$H_2(*14) + M \Rightarrow H_2 + M$		0.001	0	0	[b]
274	$H_2(*14.6) + M \Rightarrow H_2 + M$		0.001	0	0	[b]

[a] Enhanced third body efficiency ; [b] Sink reactions [32] ; f(σ) = Energy dependent cross section.

References

[1] Phelps A V and Pitchford L C 1985 *Phys. Rev. A* **31**(5) 2932–2949

[2] Campbell L, Brunger M, Nolan A, Kelly L, Wedding A, Harrison J, Teubner P, Cartwright D and McLaughlin B 2001 *Journal of Physics B: Atomic, Molecular and Optical Physics* **34** 1185

[3] Itikawa Y 2006 *Journal of physical and chemical reference data* **35** 31–53

[4] Morgan W L 1992 *Plasma chemistry and plasma processing* **12** 477–493

[5] Morgan W L URL www.lxcat.net/Morgan

[6] Janev R and Reiter D 2002 *Physics of Plasmas* **9** 4071–4081

[7] Janev R K and Reiter D 2003 *ChemInform* **34**

[8] Buckman S and Phelps A 1985 *The Journal of chemical physics* **82** 4999–5011

[9] Yoon J S, Song M Y, Han J M, Hwang S H, Chang W S, Lee B and Itikawa Y 2008 *Journal of Physical and Chemical Reference Data* **37** 913–931

[10] Marques L, Jolly J and Alves L 2007 *Journal of Applied Physics* **102** 063305

[11] Janev R and Reiter D 2004 *Physics of Plasmas* **11** 780–829

[12] Snoeckx R, Setareh M, Aerts R, Simon P, Maghari A and Bogaerts A 2013 *international journal of hydrogen energy* **38** 16098–16120

[13] Pintassilgo C, Jaoul C, Loureiro J, Belmonte T and Czerwiec T 2007 *Journal of Physics D: Applied Physics* **40** 3620

[14] Pintassilgo C and Loureiro J 2010 *Advances in Space Research* **46** 657–671

[15] Pintassilgo C, Loureiro J, Cernogora G and Touzeau M 1999 *Plasma Sources Science and Technology* **8** 463

[16] NIST Chemical Kinetics Database 2008 URL https://kinetics.nist.gov/kinetics/index.jsp

[17] Frenklach M, Bowman C, Smith G and Gardiner W 1999 *Version* **3**

[18] Zheng H and Liu Q 2014 *Mathematical Problems in Engineering* **2014**

[19] Legrand J C, Diamy A M, Hrach R and Hrachova V 1997 *Vacuum* **48** 671–675

[20] Smith G P 1999 URL http://www.me.berkeley.edu/gri_mech/

[21] Huang J and Bushe W 2006 *Combustion and Flame* **144** 74–88

[22] Huang J, Hill P, Bushe W and Munshi S 2004 *Combustion and flame* **136** 25–42

[23] Oumghar A, Legrand J, Diamy A, Turillon N and Ben-Aim R 1994 *Plasma Chemistry and Plasma Processing* **14** 229–249

[24] Tsang W and Hampson R 1986 *Journal of physical and chemical reference data* **15** 1087–1279

[25] Fincke J R, Anderson R P, Hyde T A and Detering B A 2002 *Industrial & engineering chemistry research* **41** 1425–1435

[26] Seiser R, Pitsch H, Seshadri K, Pitz W and Gurran H 2000 *Proceedings of the Combustion Institute* **28** 2029–2037

[27] Pitz W J, Naik C, Mhaoldúin T N, Westbrook C K, Curran H J, Orme J P and Simmie J 2007 *Proceedings of the combustion institute* **31** 267–275

[28] Hughes K J, Tomlin A S, Dupont V A and Pourkashanian M 2002 *Faraday Discussions* **119** 337–352

[29] Indarto A, Choi J W, Lee H and Song H K 2005 *Journal of Natural Gas Chemistry* **14** 13–21

[30] Halberstadt M L and Crump J 1972 *Journal of Photochemistry* **1** 295–305 ISSN 0047-2670

[31] Gal'tsev V E, Dem'yanov A V, Pevgov V G and Sharkov V F 1979 *Preprint IAE-3156* (Moscow: Russian Scientific Center "Kurchatov Institute")

[32] Ananthanarasimhan J and Rao L 2022 *Journal of Physics D: Applied Physics* **accepted**
